# Implementing and monitoring the right to health in breast cancer: selection of indicators using a Delphi process

**DOI:** 10.1186/s12939-023-01964-w

**Published:** 2023-07-29

**Authors:** Lisa Montel, Michel P. Coleman, Therese Murphy, Dina Balabanova, Raffaele Ciula, Dabney P. Evans, Claire Lougarre, Didier Verhoeven, Claudia Allemani

**Affiliations:** 1 London School of Hygiene and Tropical Medicine, Cancer Survival Group, Faculty of Epidemiology and Population Health, Keppel Street, London, WC1E 7HT UK; 2 Queen’s University Belfast, Belfast, UK; 3 Raoul Wallenberg Visiting Chair, Lund University, Lund, Sweden; 4 London School of Hygiene and Tropical Medicine, Faculty of Public Health and Policy, London, UK; 5 Sapienza University of Rome, Rome, Italy; 6 Hubert Department of Global Health, Emory University, Atlanta, GA USA; 7 Ulster University, Belfast, UK; 8 University of Antwerp, Antwerp, Belgium

**Keywords:** Breast cancer, Cancer control, Human rights-based approach to health, Monitoring, People-centred health system, Right to health

## Abstract

**Background:**

Women with breast cancer have different chances of surviving their disease, depending on where they live. Variations in survival may stem from unequal access to prompt diagnosis, treatment and care. Implementation of the right to health may help remedy such inequalities. The right to health is enshrined in international human rights law, notably Article 12 of the International Covenant on Economic, Social and Cultural Rights. A human rights-based approach to health requires a robust, just and efficient health system, with access to adequate health services and medicines on a non-discriminatory basis. However, it may prove challenging for health policymakers and cancer management specialists to implement and monitor this right in national health systems.

**Method:**

This article presents the results of a Delphi study designed to select indicators of implementation of the right to health to inform breast cancer care and management. In a systematic process, 13 experts examined an initial list of 151 indicators.

**Results:**

After two rounds, 54 indicators were selected by consensus, three were rejected, three were added, and 97 remained open for debate. For breast cancer, right-to-health features selected as worth implementing and monitoring included the formal recognition of the right to health in breast cancer strategies; a population-based screening programme, prompt diagnosis, strong referral systems and limited waiting times; the provision of palliative, survivorship and end-of-life care; the availability, accessibility, acceptability and quality (AAAQ) of breast cancer services and medicines; the provision of a system of accountability; and the collection of anonymised individual data to target patterns of discrimination.

**Conclusion:**

We propose a set of indicators as a guide for health policy experts seeking to design national cancer plans that are based on a human rights-based approach to health, and for cancer specialists aiming to implement principles of the right to health in their practice. The 54 indicators selected may be used in High-Income Countries, or member states of the OECD who also have signed the International Covenant on Economic, Social and Cultural Rights to monitor progress towards implementation of the right to health for women with breast cancer.

**Supplementary Information:**

The online version contains supplementary material available at 10.1186/s12939-023-01964-w.

## Background

Women with breast cancer have different chances of surviving their disease, depending on where they live. When diagnosed early and treated adequately, long-term survival is increasingly achievable [1]. At present, however, survival varies widely, both between and within countries. When such survival inequalities are avoidable through reasonable means, they constitute health inequities [2]. Such inequities are intrinsically unfair, there is a moral and public health imperative to alleviate them [3]. For those states which are a party to the International Covenant on Economic, Social and Cultural Rights (the Covenant) – the international treaty protecting the human right to health – this moral imperative becomes a legal obligation [4, 5].

A human rights-based approach to health requires a just and efficient health system, with access to adequate health services and medicines, without discrimination based on ethnicity, gender or other characteristics. However, it may prove challenging to implement and monitor the right to health, both for health policymakers and cancer specialists. This requires targeted indicators that measure whether and to what extent breast cancer care and management implement principles of the human right to health.

It is challenging to assess how well the right to health is implemented in relation to non-communicable diseases (NCDs), such as breast cancer, because of the plurality of causal factors – within and beyond the health system – and the long latency period. Years, even decades, may be needed for the first symptoms to manifest after exposure to a harmful agent. This contrasts with infectious diseases, for which the incubation period is usually much shorter (often only a few days), and the time and place of exposure to a virus can usually be located with more precision, so the cause(s) of a communicable disease can be identified with more accuracy. Given the nature of NCDs, assessing the impact of national policies on health outcomes can be complex, and may require tracking over much longer periods. Such assessments must include a range of economic and social variables, as opposed to being limited to individual behaviours or exposure to a particular agent [6].

Increasing attention is now being paid to measurement of progress towards implementation of the right to health. For example, since the publication of General Comment 14 – the interpretative guidance issued by the body responsible for implementation and monitoring of the Covenant [5] – efforts have focused on developing new methods, including the construction of indicators to measure implementation [5]. Landmark studies conducted by human rights experts include a list of 72 indicators, [7] the OPERA framework, [8] and the social and economic rights fulfilment index (SERF index) [9]. So far, these attempts have focused on measuring implementation of the right to health in general, [7] and of the right to health applied to maternal health, [10, 11] infectious diseases such as tuberculosis, [12] access to essential medicines, [13] or focusing on methods to determine what level of rights fulfilment is achievable for any given level of resources [9]. To our knowledge, no set of indicators has been developed in relation to cancer. Here, we aimed to construct a set of indicators of implementation of the right to health that can inform breast cancer care and management in women.^1^

## Method

### A preliminary list of right-to-health indicators for breast cancer

We constructed a preliminary list of 151 indicators to measure implementation of the right to health in breast cancer care and management. We followed a three-step process. First, we identified existing right-to-health indicators and adapted them to breast cancer. Second, we constructed a novel set of indicators to measure the extent to which the right to health is applied to breast cancer care and management, and the extent to which breast cancer patients experience the right to health throughout their cancer journey, from screening, early diagnosis, treatment and palliative care to end-of-life care. Finally, we organised the 151 indicators using a combination of established frameworks designed to assess health systems and the right to health [7, 14, 15].

#### Adapting existing right-to-health indicators to breast cancer

##### The right to health in scientific databases

To understand the extent to which the principles underlying the right to health are perceived and used in the public health community, a scoping review of the right to health was conducted by examining publications between 2000 and 2021 in five scientific databases: Embase, Global Health, Medline, Open Grey, and Dissertations and Theses Global, using the keywords “right to health”, “human rights-based approach to health” and “indicators” or “framework.” [16]. The findings of the scoping review informed the design of our preliminary list of indicators.

The most significant attempt to assess implementation of the right to health was a list of 72 indicators published in 2008 by authors including the first UN Special Rapporteur on the right to health [7]. These indicators were constructed following a rigorous method that included:

- An assessment of all sources of international law mentioning the right to health;
- A review of all existing projects at the time from international agencies such as the World Health Organisation (WHO) and the World Bank;
- A consultation with experts including academics, UN bodies, national and local non-governmental organisations, health practitioners, lawyers, economists and anthropologists.

The indicators were selected on strict criteria: scientific robustness, usefulness, representativeness, understandability and importance.

We compiled a list of indicators relevant to the right to health applied to breast cancer care and management. These indicators, retrieved from the scoping review, included some of the 72 indicators described above. We adapted these indicators to the context of breast cancer. For example, the indicator on a national health plan was adapted to become an indicator for the availability of a national cancer strategy. Then, we added all relevant indicators for a rights-based approach to breast cancer care and management from the Organisation for Economic Co-Operation and Development (OECD), such as the percentage of total health expenditure spent on cancer care. We chose the OECD for their collaborative work with international experts on the development of indicators to measure cancer care and management across the entire health system, notably through their Health Care Quality Indicators project and their Health at a Glance publications [17].

##### The right to health in legal databases

We also conducted three nested scoping reviews of right-to-health indicators in legal databases: human rights indicators, economic and social rights indicators,^2^ and right-to-health indicators. We looked in the three main legal databases: HeinOnline, Westlaw and LexisNexis. We extended our search from 1966 to 2021 because the Covenant was first opened for signatures in 1966. The aim was to identify previous attempts to assess implementation of the right to health or of economic and social rights, notably to understand how the legal principles that underpin this category of rights were translated into measurable indicators. This is because under the Covenant, states must realise the right to health “progressively” and “to the maximum of their available resources” [4]. States also have immediate obligations, such as implementing the right on a non-discriminatory basis and taking concrete steps towards progressive realisation [4, 18]. Indicators are a key tool to assess the core minimum and progress of economic and social rights over time for any given level of resources [9, 19].

We did not select any indicators from the legal literature because these studies tended to use conventional public health indicators, such as the mortality rate of children under five, or life expectancy. Legal studies were informative on translation of the principles of progressive realisation, core minimum obligations, maximum available resources and non-discrimination into measures.

#### Constructing novel right-to-health indicators throughout the breast cancer pathway

##### Principles from international human rights law sources

Before new indicators could be constructed, we determined the content and scope of the right to health in relation to breast cancer [20, 21]. We reviewed international human rights law documents protecting the right to health, notably Article 12 of the Covenant [4] and General Comment 14 [5]. Provisions from other instruments that protect specific population groups were also included [22–24]. This assessment of international human rights law instruments complemented the scoping review of the right to health in legal databases to ensure that all legal principles underlying the right to health would be included in the preliminary list of indicators.

Other procedural principles also emanate from the obligations of states, such as participation of affected populations in important decisions, and the transparency and accountability of national institutions [18, 25, 26]. Further, General Comment 14 enunciates the “AAAQ” framework,^3^ namely the availability, accessibility, acceptability and quality of health services, goods and medicines [5]. The right to health includes underlying determinants too, such as access to potable water, clean sanitation, adequate housing and safe working conditions [5].

We attempted to translate the core principles of the right to health into measurable indicators, focusing on breast cancer. The principles selected to construct the preliminary list of indicators are outlined in Table 1.

**Table 1:** Principles of the right to health used to inform the construction of indicators for breast cancer management and care, adapted from references #5 and #7

Principle	Definition	International human rights law sources
Availability Accessibility Acceptability Quality (AAAQ)^a^	All health services, goods and facilities shall be available, accessible, acceptable and of good quality (AAAQ). The precise nature of these elements will depend on the conditions prevailing in a particular state Available: functioning public health and health-care facilities, goods and services, as well as programmes, have to be available in sufficient quantity within the state party. The precise nature will vary depending on numerous factors, including the state party’s development level Accessible: health facilities, goods and services have to be accessible to everyone without discrimination, within the jurisdiction of the state party. It includes: non-discrimination; physical accessibility; affordability and information accessibility Acceptable: all health facilities, goods and services must be respectful of medical ethics and culturally appropriate as well as being designed to respect confidentiality and improve the health status of those concerned Good quality: health facilities must be scientifically and medically appropriate and of good quality. This requires skilled medical personnel, scientifically approved and unexpired drugs and hospital equipment, safe and potable, and adequate sanitation	General Comment 14
Accountability^b^	[T]he right to health brings with it the crucial requirement of accessible, transparent and effective mechanisms of monitoring and accountability. Those with right-to-health responsibilities must be held to account in relation to the discharge of their duties, with a view to identifying successes and difficulties; so far as necessary, policy and other adjustments can then be made. Examples of accountability mechanisms are: 1.Judicial, e.g. judicial review of executive acts and omissions 2. Quasi-judicial, e.g. (…) human rights treaty-bodies 3. Administrative, e.g. human rights impact assessment 4. Political, e.g. parliamentary committees 5. Social, e.g. civil society movements The accountability mechanism should exist at the national, regional (if available) and international levels. Rightsholders are also entitled to effective remedies when duty-bearers have failed to discharge their right to health obligations. These remedies may take the form of restitution, rehabilitation, compensation, satisfaction or guarantees of non-repetition	General Comment 14 Limburg Principles Maastricht Guidelines
Core obligations^c^	In general comment No. 3, the Committee confirms that States parties have a core obligation to ensure the satisfaction of, at the very least, minimum essential levels of each of the rights enunciated in the Covenant, including essential primary health care. Read in conjunction with more contemporary instruments, such as the Programme of Action of the International Conference on Population and Development, the Alma-Ata Declaration provides compelling guidance on the core obligations arising from article 12. Accordingly, in the Committee’s view, these core obligations include at least the following obligations: a) To ensure the right of access to health facilities, goods and services on a non-discriminatory basis, especially for vulnerable or marginalized groups; b) To ensure access to the minimum essential food which is nutritionally adequate and safe, to ensure freedom from hunger to everyone; c) To ensure access to basic shelter, housing and sanitation, and an adequate supply of safe and potable water; d) To provide essential drugs, as from time to time defined under the WHO Action Programme on Essential Drugs; e) To ensure equitable distribution of all health facilities, goods and services; f) To adopt and implement a national public health strategy and plan of action, on the basis of epidemiological evidence, addressing the health concerns of the whole population; the strategy and plan of action shall be devised, and periodically reviewed, on the basis of a participatory and transparent process; they shall include methods, such as right to health indicators and benchmarks, by which progress can be closely monitored; the process by which the strategy and plan of action are devised, as well as their content, shall give particular attention to all vulnerable or marginalized groups The Committee also confirms that the following are obligations of comparable priority: a) To ensure reproductive, maternal (prenatal as well as post-natal) and child health care; b) To provide immunization against the major infectious diseases occurring in the community; c) To take measures to prevent, treat and control epidemic and endemic diseases; d) To provide education and access to information concerning the main health problems in the community, including methods of preventing and controlling them; e) To provide appropriate training for health personnel, including education on health and human rights	General Comment 14, paras 43 and 44 General Comment 3
Legal recognition^d^	Legal recognition of the right to health is the first step towards its implementation. Legal recognition means that states must ratify human rights treaties recognising the right to health, such as the ICESCR, and incorporate the right to health into their national constitution	Article 12 ICESCR
Maximum available resources	States must devote the maximum available resources to the progressive realisation of economic, social and cultural rights. “Resources” are understood to include financial, natural, human, technological, and informational resources.^7^ States in a position to assist should provide resources to other states in need so that they can realise the right to health of their populations. In turn, states with scarce resources have an obligation to ask the international community for assistance	Article 2.1 ICESCR General Comment 3 General Comment 14
Non-discrimination^e^	The principle of non-discrimination seeks to guarantee that human rights are exercised without discrimination of any kind based on race, colour, sex, language, religion, political or other opinion, national or social origin, property, birth or other status such as disability, age, marital and family status, sexual orientation and gender identity, health status, place of residence, economic and social situation Non-discrimination and equality of men and women before the law are not subject to the principle of progressive realisation; they must be implemented immediately	Article 2.1 ICESCR Article 3 ICESCR Limburg Principles Maastricht Guidelines General Comment 14 General Comment 20
Participation^f^	Active and informed participation of individuals and communities in decision-making that has a bearing on their health	General Comment 14 Limburg Principles General Comment 20
Responsibility to respect, protect, fulfil^g^	States have duties to respect, protect and fulfil the right to the highest attainable standard of health. These duties are equally applicable to medical care and the underlying determinants of health. The obligation to respect requires States to refrain from interfering directly or indirectly with the enjoyment of the right to health. The obligation to protect requires States to take measures that prevent third parties from interfering with the right to health. Finally, the obligation to fulfil requires States to adopt appropriate legislative, administrative, budgetary, judicial, promotional and other measures towards the full realisation of the right to health	General Comment 14

^a^Definition from the glossary in Backman et al. (webappendix): Backman G, Hunt P, Khosla R, et al. Health systems and the right to health: an assessment of 194 countries. *Lancet* 2008; **372**(9655): 2047–85

^b^Ibid

^c^Definition from UN Committee on Economic Social and Cultural Rights. General Comment No. 14: The Right to the Highest Attainable Standard of Health (Art. 12 of the Covenant). E/C12/2000/4; 11 August 2000

^d^Definition from UN Committee on Economic Social and Cultural Rights. General comment No. 20: Non-discrimination in economic, social and cultural rights (art. 2, para. 2, of the International Covenant on Economic, Social and Cultural Rights). E/C12/GC/20; 2009

eDefinition from the glossary in Backman et al. (webappendix): Backman G, Hunt P, Khosla R, et al. Health systems and the right to health: an assessment of 194 countries. *Lancet* 2008; **372**(9655): 2047–85

fDefinition from the glossary in Backman et al. (webappendix): Backman G, Hunt P, Khosla R, et al. Health systems and the right to health: an assessment of 194 countries. *Lancet* 2008; **372**(9655): 2047–85

^g^Ibid

##### Translation of right-to-health principles into measurable indicators of the breast cancer pathway

Novel indicators were constructed, based on the key principles of a human rights-based approach to health throughout the breast cancer journey: the availability, accessibility, acceptability and quality of health services, goods and medicines [5]. Indicators were also constructed using the principle of informed and active participation of breast cancer patients in decisions that affect them, such as the decision to undergo a full mastectomy, or a choice between chemotherapy treatments with due regard to their side effects and their impact on survival. The principle of accountability was also drawn upon, i.e., accountability of health institutions and the state at the national, regional and local level, offering appropriate remedies in case of any breach of patients’ right to health.

We referred to international clinical guidelines on breast cancer screening, diagnosis and treatment published by the European Society for Medical Oncology [27, 28] and the American Society of Clinical Oncology (ASCO) [29]. We also consulted standards and benchmarks published by WHO, [14] OECD, [30] and the International Atomic Energy Agency for their work on access to radiotherapy [31]. In order to ensure that we adopted a people-centred approach, we used the recently published guide on the optimal breast cancer pathway [32]. Finally, we consulted international studies indicated by experts that we approached for this study. These articles assessed areas of interest, for instance national cancer plans or the workload and job satisfaction in medical oncology [33, 34].

#### Anchoring the indicators into existing frameworks

**Fig. 1:**
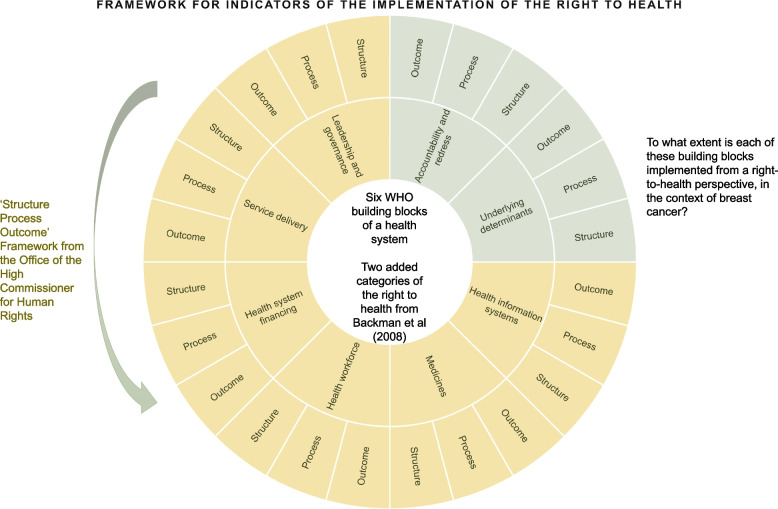
Framework for indicators of the implementation of the right to health

Our preliminary list was designed to be a coherent set of indicators that relate to a comprehensive conceptual framework. They drew on the “structure, process, outcome” framework of the Office of the High Commissioner for Human Rights (Fig. 1) [15]. Under this framework, structural indicators provide information on whether laws and policies in a country reflecting its commitment to realising a particular human right actually exist. Process indicators identify measures that the state is taking to implement the law or policy in question. They provide a check that the political commitment is being delivered, rather than an empty promise. Finally, outcome indicators offer information on the potential impact of such laws or policies in practice. When chosen carefully, the structure, process and outcome indicators establish links between the existence and implementation of laws, and human rights outcomes – even if causality cannot be established. They offer an opportunity to reflect on what policy or programme works in a given context, i.e., improves health outcomes.

With a view to anchoring our work into practice, we designed indicators for each of the six building blocks of a health system defined by the WHO: leadership and governance; service delivery; health system financing; health workforce; medical products, vaccines and technologies; and health information systems. For each building block, we aimed to answer the question: “To what extent is this building block implemented from a right-to-health perspective, in the context of breast cancer?”.

Finally, we added two categories that are specific to the right to health from the landmark 2008 study: “action on the underlying determinants of health” and “accountability and redress” [7]. This ensured that all aspects of the right to health were considered when constructing the indicators. We deliberately omitted a category on international assistance and cooperation because we focused on the national and organisational approach to breast cancer care and management in low-, middle- and high-income settings. However, we argue that, in principle, international cooperation and assistance should be measured as a component of the right to health.

The preliminary list of 151 indicators of the implementation of the right to health for breast cancer included 23 existing and 128 novel indicators (Appendix 1). The aim of the Delphi study was to select the most relevant indicators with the structured input of experts who had a range of disciplinary backgrounds and expertise, using an interdisciplinary approach.

### Delphi method

The Delphi technique is commonly used to develop healthcare quality indicators [35]. A robust Delphi method is characterised by five criteria: anonymity of participants, to control for dominant individuals; iteration of rounds to reach a consensus; controlled feedback to allow each participant to revise their answers against those of the group; statistical group response to determine when consensus is reached, and finally, expert input [35–37]. These principles guided our exercise.

Participants were invited in December 2020 and the study was conducted from January to September 2021. Participants were asked to score each of the 151 indicators using a Likert scale from 1 to 5, 1 being “strongly disagree” and 5 being “strongly agree.” Indicators for which early consensus was reached were excluded from future rounds. Anonymous feedback was provided as a summary for the group, and separately for each participant, to stimulate debate and encourage consensus. Following best practice, consensus to select an indicator was defined as at least 80% of participants giving a score of 4 or 5 [35]. Conversely, consensus to reject an indicator was defined as at least 80% of participants giving a score of 1 or 2. For each indicator, there was an option to add a comment. However, for practical reasons, we asked that this option should be used only if participants felt that the indicator was controversial, complex or needed to be reworded. The comments were merged and shared with the group as feedback, to inform selection of indicators in the next round.

**Fig. 2:**
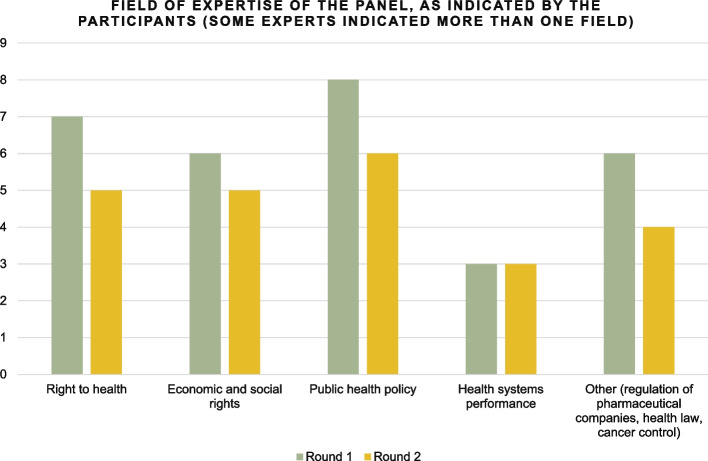
Fields of expertise of panel members

A heterogeneous group of experts was invited to participate. They came from a wide range of disciplines: human rights, including economic and social rights, health systems research, public health policy, cancer control, regulation of pharmaceutical companies, including through patient advocacy, and health law (Fig. 2). Two techniques were used to maximise responses to the invitations. First, the study was endorsed by the first UN Special Rapporteur on the right to health. Second, an initial pool of influential experts was invited in advance of the wider group, to stimulate participation by other experts. The names of these first few experts were mentioned in the invitation to the wider group, to underline the legitimacy of the study. Thirty-one experts were invited by email, twenty experts agreed to participate, six refused and five did not reply, despite two reminders. Of the 20 experts who agreed to participate, 13 replied to the first round of selection, and 10 to the second round. Of the 13 experts who responded, four had less than 10 years of experience, five between 10 and 25 years, and four had more than 25 years of experience. Ten participants were based in Europe and three in North America. Those who responded to both rounds were invited to co-author this paper. They were not invited until after the study had officially ended, to safeguard anonymity during the selection process. The number of experts invited, technique used to increase chances of participation and processes by which the study were conducted were informed by a meta-analysis of Delphi methods used in previous studies [35].

For each of the 23 existing indicators, participants were simply asked to score how much they agreed or disagreed that it could be used to measure implementation of the right to health in breast cancer care and management. For the 128 novel indicators, participants were asked to score the extent to which the indicator would be scientifically robust, useful, representative, understandable, and important (Table 2) [7]. Feasibility and data availability were not taken into account to select indicators. This is because the final selection was intended to be an *ideal* set of indicators, which could in principle be used to design robust monitoring systems and to stimulate data collection. In practice, different sub-sets of these indicators could be used, depending on which actors use them and their aim in using them.

**Table 2:** Criteria used to select right-to-health indicators

Criterion	Definition
**Scientific robustness**	Indicators are reliable (i.e., they provide stable results across various populations and circumstances) and valid (i.e., they measure what they are intended to measure)
**Usefulness**	Indicators evaluate areas that need improvement and require prioritisation, or right-to-health principles, as opposed to what data are available
**Representativeness**	Indicators are based on observed data as opposed to estimates from models that rely on assumptions
**Understandability**	The measures are clear and understandable by policymakers
I**mportance**	Indicators reflect important elements of the right to health or a human rights-based approach to breast cancer

Definitions of these criteria are from E Nolte, M McKee, S Wait. Describing and evaluating health systems. In A Bowling, S Ebrahim (Eds.), Handbook of health research methods, Open University Press, New York (2006), pp. 12–43

A document detailing all 151 indicators was attached to the questionnaire, with a glossary of terms (Appendices 1 and 2). Participants could refer to this document to verify whether any indicator proposed was already in use in other global agencies, or completely novel, and to validate the data sources that were being proposed to populate this indicator. This document also included a short rationale on why the indicator was relevant to the right to health as applied to breast cancer.

The list of indicators was updated after analysis of responses in the first round (Table 3). Indicators were then included in the second round only if there was no consensus in the first round. Some indicators were reformulated in the light of experts’ comments, and three indicators were added. Each indicator was provided to participants with anonymous group feedback alongside their own responses in round 1 (Table 4).

**Table 3:** Example of the questionnaire for one indicator in the first round

Indicator	1	2	3	4	5	Comment
Does the state’s Constitution protect the right to health?

**Table 4:** Example of the questionnaire in round 2 for one indicator that did not reach consensus in the first round, with individual and group feedback

	**ROUND 1**									**ROUND 2**					
**No consensus in round 1**	**Your responses**					**Group responses**			**Group feedback**	**Your responses**					**Your feedback**
**Indicator #7**	1	2	3	4	5	1&2	3	4&5		1	2	3	4	5	
In the national cancer plan, there is a strategy to implement a multidisciplinary care for breast cancer				x		36%	0%	64%	"Large country variations, difficult to standardise and apply consistently across countries." "Might depend on country capacity."						

Existing indicators for which a disaggregated form was novel were considered as novel (indicators #25, 26, 27, 40, 51 and 86). Disaggregated versions of an indicator were only selected if the original indicator was also selected.

Incidence and survival for breast cancer are not included in the table as indicators of the implementation of the right to health in breast cancer care and management. This is because incidence and survival are outcome metrics of whether implementation of a national cancer strategy or plan actually changes the number of people who get sick and the proportion of those people who die from the disease.

## Results (Table 5)

**Table 5:** List of 54 selected right-to-health indicators in breast cancer care and management

Health service delivery
**Structural indicators**
1. In the NCP or NCDP there is a strategy to implement population-based breast cancer screening
2. In the NCP or NCDP there is a strategy to implement access to radiotherapy
3. In the NCP or NCDP there is a strategy to implement palliative/supportive care
4. In the NCP or NCDP there is a strategy to implement survivorship care
5. In the NCP or NCDP there is a strategy to implement end-of-life care
**Process indicators**
6. There is a referral system in place from primary care to oncology services
7. There is a specified maximum waiting time between diagnostic suspicion in primary care and the first appointment with an oncologist
8. There is a specified maximum waiting time between the confirmed diagnostic and the first appointment for treatment
9. The national diagnosis guidelines involve pathological evaluation in line with ESMO or ASCO recommendations
10. The number of radiotherapy units is at least as high as the optimal threshold set by the IAEA (one radiotherapy unit per 500,000 population)
Disaggregated by geography
11. There is a referral system in place from the breast unit to psychological care
Disaggregated by geography
12. There is a trained member of staff acting as patient navigator in the breast unit
Disaggregated by geography
**Outcome indicators**
13. Proportion of suspected breast cancer patients with a first consultant appointment within 2 weeks of primary care referral
14. Proportion of women with advanced breast cancer (stage IV) at diagnosis
Disaggregated by wealth quintile and ethnicity
15. Proportion of breast cancer patients forgoing or postponing care because of limited availability
Disaggregated by wealth quintile and ethnicity
16. Proportion of breast cancer patients forgoing care because of affordability
Disaggregated by wealth quintile and ethnicity
17. Proportion of breast cancer patients who receive palliative care
Disaggregated by wealth quintile and ethnicity
18. Proportion of women terminally ill with breast cancer who receive end-of-life care
Disaggregated by wealth quintile and ethnicity
**Health system financing**
**Structural indicators**
19. The NCP or NCDP addresses costs of implementation of the breast cancer strategy
20. Share of government spending and out-of-pocket payment out of the total spending on health per capita
21. The state has a social health insurance system
**Process indicators**
22. The social health insurance system covers diagnostic services for breast cancer (i.e., biopsy, mammogram and ultrasound)
23. The social health insurance system covers breast cancer treatment (i.e., hormone therapy and chemotherapy)
24. The social health insurance system covers radiotherapy for breast cancer
25. The social health insurance system covers palliative care for breast cancer
**Outcome indicators**
26. Proportion of costs covered for breast cancer care by the social health insurance system
**Medicines**
**Structural indicators**
27. There is an official national medicines policy to provide access to essential medicines
28. The NCP or NCDP mentions breast cancer medicines included in the WHO Essential Medicines List
**Process indicators**
29. Proportion of breast cancer medicines included in the WHO Essential Medicines List that are available in the country and covered by public funding
**Outcome indicators**
30. Proportion of hospitals with palliative medicines shortage
Disaggregated by geography
**Health workforce**
**Structural indicators**
31. The state has a national health workforce strategy
**Process indicators**
None selected
**Outcome indicators**
32. Prevalence of certified oncologists per 1,000,000 population
Disaggregated by geography
**Health information systems**
**Structural indicators**
33. The state law requires informed consent to treatment and other health interventions
34. The NCP or NCDP protects the right to seek and receive health information
35. The NCP or NCDP addresses communication of information throughout the pathway of care for breast cancer, from screening through to referral, diagnosis, treatment options and palliative care
36. The NCP or NCDP addresses the needs of patients from culturally and linguistically diverse backgrounds
37. The NCP or NCDP addresses participation of patients in decisions that affect them
**Process indicators**
38. The personnel in the breast unit are trained to communicate information on diagnosis and treatment options, including side effects and survival, repeatedly, verbally and in writing, in a comprehensive and easily understandable form to patients
39. The personnel in the breast unit presents all options available to the patient beside a mastectomy, and explains in an easily understandable way psychological distress that may be associated with a mastectomy
40. The personnel in the breast unit discusses breast reconstruction techniques individually taking into account anatomic, treatment- and patient-related factors and preferences
41. The breast cancer unit uses trained interpreters when communicating with patients from culturally and linguistically diverse background
42. The breast unit has a participatory process in place to include patients into decisions that affect them individually
**Outcome indicators**
43. Proportion of breast cancer patients who feel they have received sufficient, comprehensive and easily understandable information, including on treatment side effects and survival, to be involved in decisions about their care
Disaggregated by wealth quintile, ethnicity, and language
44. Proportion of breast cancer patients who feel they have been involved in decisions about their care
**Governance and leadership**
**Structural indicators**
45. The Constitution, Bill of Rights, or other statute recognises the right to health
46. The NCP or NCDP addresses accountability of the state and health institutions
47. The NCP or NCDP includes a protection against discrimination
48. The NCP or NCDP includes an explicit commitment to universal access to cancer services and treatment
49. The NCP or NCDP includes a set of targets and progress indicators specific to breast cancer
**Process indicators**
None selected
**Outcome indicators**
None selected
**Underlying determinants of breast cancer**
**Structural indicators**
None selected
**Process indicators**
None selected
**Outcome indicators**
None selected
**Accountability and redress**
**Structural indicators**
50. There is an accessible pre-judicial mechanism to lodge complaints alleging breach of obligations connected to the right to health
**Process indicators**
51. The breast unit has a formal complaints mechanism for patients
**Outcome indicators**
None selected
**Additional indicators suggested in round 1 and selected in round 2**
52. Proportion of the population at risk participating in the screening programme
53. Prevalence of certified nurses per 1,000,000 population
54. The State has ratified key human rights treaties recognising the right to health

**Fig. 3:**
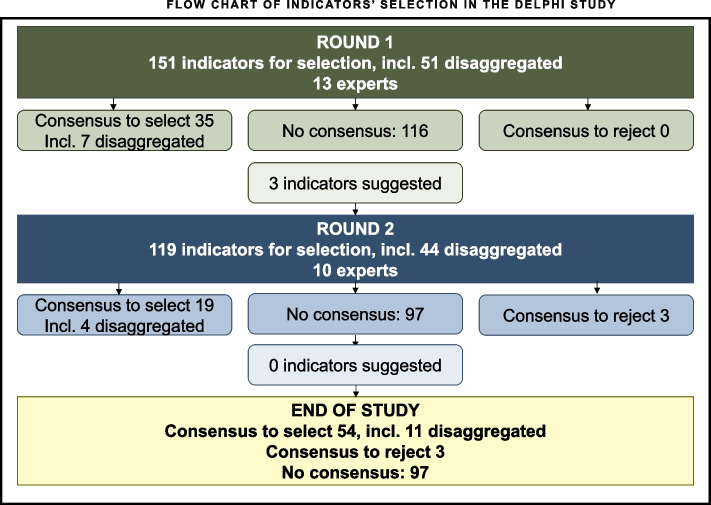
Flow chart of indicators’ selection in the Delphi study

After two rounds of selection, 54 (36%) of 151 the indicators achieved consensus for selection and three (2%) were rejected (Fig. 3). No consensus was reached to select or reject the remaining 97 (64%) indicators. In the first round, 80% or more of the panel agreed to select 35 indicators, including seven with disaggregated data. There was no consensus to reject any indicator. Three indicators suggested by the panel were added to the second round for selection. Some experts suggested rewording certain indicators to add clarity, as indicated in Appendix 1 and 3. In the second round, the panel agreed to select 19 more indicators, including four indicators disaggregated by ethnicity. The panel also agreed to reject three indicators. No further indicators were added.

**Fig. 4:**
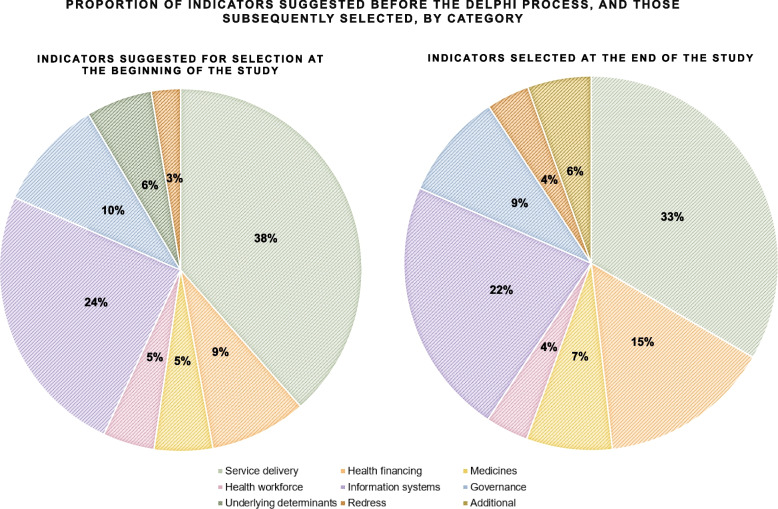
Proportion of indicators suggested before the Delphi process, and those subsequently selected, by category

Thirty (56%) of the 54 indicators selected belong to two categories: health service delivery and health information systems (Fig. 4). These indicators evaluate the availability, accessibility, acceptability and quality (AAAQ) of health facilities and services, as well as the right of patients to seek and receive high-quality information to be empowered and participate in decisions that affect them.

**Fig. 5:**
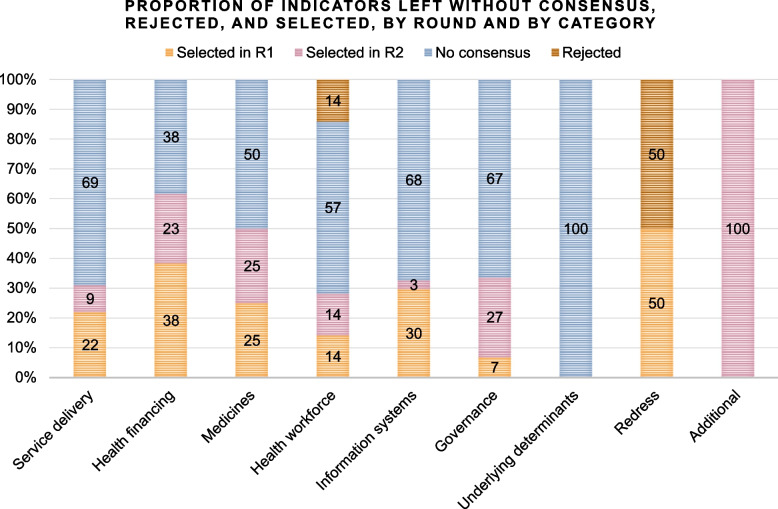
Proportion of indicators left without consensus, rejected, and selected, by round and by category

At least 30% of indicators were selected in each of the categories, except for the underlying determinants (Fig. 5). The largest proportion was from the category “health systems financing” (62% of indicators in that category were selected), followed by “access to essential medicines” (50%). Two of the four indicators in the “accountability and redress” category were also selected. Experts agreed that disaggregated information should also be collected for 11 indicators.

Novel indicators designed for this study comprised 85% of the indicators selected in the Delphi process (46 out of 54).

## Discussion

### Indicators for which there was a consensus

Thirty (56%) of the 54 indicators of a human rights-based approach to breast cancer selected by the panellists were from two of the eight categories: “health service delivery” and “health information systems”. These categories contained 95 (63%) of the 151 indicators they were asked to assess. This suggests that components of the right to health in these categories may lend themselves better to measurability, in particular the AAAQ of health services and facilities. In addition, eight (15%) and four (7%) of the 54 indicators selected in “health systems financing” and “access to essential medicines” respectively suggest the importance of funding allocation and access to essential medicines when assessing a human rights-based approach to breast cancer care and management. Interestingly, not a single indicator was selected from the category “underlying determinants of health”, although this category has been regarded as an essential feature of the application of right to health in health systems [7]. The link between the determinants and outcomes of breast cancer may be too remote to be assessed. Whilst these determinants are an integral part of the right to health, measuring them may give an indication of the overall effectiveness of the health system rather than its effectiveness for a single condition.

Similar to the list developed in 2008, panellists selected indicators that evaluate formal state commitments to the right to health, universal access to cancer services, and to enacting a national cancer plan. Legal recognition of the human right to health is the first step to implementing it [7]. Research suggests that signature of the Covenant, the main treaty that recognises the right to health, is associated with stronger constitutional protection at the national level [38].

Other indicators selected were mostly novel. The selection stressed the importance of having a functioning population-based breast cancer screening programme, an efficient system of diagnosis, and a referral system to financially accessible cancer treatment. When breast cancer is detected and treated early, the chances of survival are higher [39–41]. Palliative care, quality of life (survivorship) and end-of-life care were selected to be measured as components of the right to health, including the availability and financial accessibility of palliative medicines for everyone. Likewise, many indicators on the AAAQ of health facilities, medicines and services were selected by the panel, including radiotherapy, chemotherapy and hormone therapy, as well as the availability of key health personnel.

The panellists selected many indicators that put forward the dignity of breast cancer patients, their right to seek and access quality information, as well as their right to take part in decisions that affect them. Outcome indicators were selected – e.g. a survey of patients – showing the importance attached by the panellists to patients’ perceptions on how information about their disease is communicated to them and whether they feel they are involved in health decisions. These indicators stress the need to adopt and monitor a “people-centred approach” to breast cancer care and management.

Although it was implicit, the “people-centred approach” to a health system was not discussed in 2008 [7]. This reveals an important human rights-based aspect that has developed in health system research since then. This concept has been discussed in several disciplines, from health policy to medicine and nursing, but it has not been uniformly defined [42]. Common themes include that the person should be at the centre of health strategies and treated with respect and dignity, and that their needs, wishes and preferences should be taken into account [42]. This approach underpins the empowerment and participation of patients, their families and communities in decisions that affect them, [42] which are key procedural principles of the right to health [5]. Human rights-based approaches to health, and before them the health and human rights movement, have placed emphasis on the dignity of the individual, the right to information about one’s health and the right to give free and informed consent to health interventions [43, 44]. The 2008 study on right-to-health indicators did place people at the centre of the right-to-health features of health systems, despite never referring to the term “people-centred”. There is considerable overlap between people-centred approaches and the right to health, despite the bodies of literature on these two domains remaining largely separate [7, 42].

Interestingly, the experts agreed to reject two indicators to evaluate the number of court cases on cancer that had been litigated in a given year, as well as the proportion of class actions among those cases. Accountability was listed as a right-to-health feature of health systems in the 2008 landmark article, suggesting that thinking about this aspect of the right to health may have evolved since then. Accountability of the state and state actors through judicial and non-judicial processes are both critical components of the right to health [5, 45].

However, these processes may sometimes be implemented in a way that perpetuates health inequalities. First, those able to claim their right to health through the judicial system, who can thus afford a lawyer, are likely to be wealthier than those who are not able to claim their right [46, 47]. Individual litigation cases may therefore result in inequitable spending on the health system. Second, judges may overstep their mandate to assess compliance with the law, by deciding how, or for whom, public funds should be spent, instead of funds being allocated to health priorities based on scientific evidence [46, 47]. For example, researchers found that the NHS Cancer Drugs Fund, which was created in the UK in 2010 to improve access to cancer drugs, did not result in cost-efficient added value to patients [48]. These examples strengthen interpretation of the principle of accountability as being a “fair and reasonable process to identify what works, so it can be repeated, and what does not, so it can be revised” rather than an individual right to have access to cancer treatment [7].

Finally, the panellists selected disaggregated sub-sets of information for many indicators. Disaggregation of sources of information exposes patterns of discrimination based on age, sex, ethnicity, language, place of residence, income, or any other ground for which data are systematically collected. Disaggregating information by wealth quintile or geography may reveal discrimination on social status. The panellists’ selection of indicators derived from disaggregated information shows that they judged these more detailed indicators as critical in assessing the right to health, in particular the principle of non-discrimination on grounds of age, sex, etc. This is consistent with the literature on human rights indicators which overwhelmingly recommends the use of disaggregated information if and when possible [8, 10, 49].

### Indicators which did not reach a consensus

The 97 indicators for which there was no consensus are diverse, but five characteristics may be observed. First, some of these indicators related to the health system generally, which means that the link with breast cancer may have been considered too remote. For instance, indicator 81: “The state law includes provision for adequate remuneration of doctors and nurses, including oncologists.” Existing indicators were meant to enable evaluation of the implementation of the right to health in health systems, so they may not be well suited to measuring its implementation in relation to the management and care of a specific condition, such as breast cancer.

Second, some of these indicators related to control of the private sector by the state. For instance, indicator 125: “The state has transparent rules on lobbying, including a public lobbying register.” Private companies are not bound by international human rights law,^4^ but a state does have the duty to protect its citizens against action from third parties, including private companies, that may impede the realisation of their right to health [5].

Third, some of these indicators may be too detailed (e.g., indicator #111^5^), which does not lend itself well to accuracy and measurability. Likewise, some of these indicators suggest that the right to health should not be too broad, such as methods to relieve stress and side-effects from treatment through music therapy, meditation, stress management, yoga, relaxation, and massage (indicators #30 and 31). Whilst these methods are recommended by ASCO for optimal care of breast cancer patients, the panellists felt that states should not be obligated to procure them under the right to health. This reinforces the principle of progressive realisation of the right to health and the limit on states’ resources.

Fourth, many indicators for which there was no consensus were outcome indicators. For example, the lack of consensus on self-reported satisfaction of breast cancer patients (indicators #51, 53, 54, 55, 119) may be seen as surprising given that patient-reported outcome measures (PROMs) have gained considerable emphasis in the last 10 years [17]. Nevertheless, the panellists may have considered such indicators to lack accuracy and comparability.

Finally, there was a surprising lack of consensus for some indicators that would enable evaluation of effective measures to prompt diagnosis, such as indicators on breast self-examination (#96, 101, 121, 122), or on screening of women at higher risk of developing breast cancer because of their family history (#2, 15, 17, 18, 41). For some indicators, the lack of consensus may be sub-optimal, because of the short duration of the study or the diversity of expertise among the panellists.

We decided to end the study after two rounds because many of the indicators were too similar to one another, and the 54 indicators selected would provide enough information to monitor implementation of the right to health in breast cancer care and management. We considered that a larger list of indicators would be impracticable, and would discourage implementation of the right to health and its monitoring.

Fifty-five indicators to evaluate a single health condition are too many to be manageable by international human rights monitoring bodies, such as the International Committee on Economic, Social and Cultural Rights (the Committee). These bodies are not only required to assess the implementation of right-to-health features in national health systems; they must also evaluate whether states dedicate the maximum of their available resources to realising the right to health for everyone, and make adequate progress over time [4]. This means that the care and management of breast cancer patients must be evaluated alongside hundreds of other health conditions, including fair prioritisation and allocation of resources, as well as implementation of other economic and social rights. Nevertheless, states that report their progress to the Committee could use a sub-set of these indicators, depending on the policy area of interest, to show progress on implementation of the right to health for women with breast cancer. As the most common cancer in women, this approach could constitute a concrete example of how member states are tackling implementation of the right to health in relation to a major non-communicable disease.

The indicators selected in this study could also assist health policymakers and cancer management specialists to implement principles of the right to health in their work, and then to monitor their implementation. For example, these indicators may inform healthcare professionals on what service or what aspects of care they should prioritise to implement their patients’ right to health. They also provide measures by which progress in implementing right-to-health features in breast cancer care and management may be monitored over time. Whilst the indicators are universal, the standards of implementation of the right to health must be set nationally, or locally, to account for resource constraints and other local conditions [19].

Indicators that were not selected by the panellists should not be ignored, however. We encourage further research into specific areas to assess components of the right to health in more detail. For instance, indicators on the satisfaction of women with the care that they received may be further refined and selected to assess the AAAQ of breast cancer care.

Despite implementing the Delphi method, this study has several limitations. First, while 10 of the 13 panel members had expertise in the right to health, public health policy, health law, or cancer control, only three had expertise in health systems monitoring or health statistics. Second, the study ended after two rounds, primarily due to time constraints. If further rounds had been conducted, more indicators might have been selected or rejected. The 97 indicators for which no consensus was reached may be subject to further research. However, we argue that this initial list can still be used by policymakers at national level to assess progress in particular areas of the right to health, according to their key policy priorities. Third, not all experts voted on all indicators, which means that some indicators were selected with fewer than 13 votes in the first round, and fewer than 10 votes in the second round. Even so, six votes was the smallest number of experts agreeing to select or reject an indicator.

Some of the indicators selected may be readily available from national health statistics or international databases such as those of OECD or the World Bank. However, data enabling estimation of most selected indicators may not be systematically collected, especially in a disaggregated form. For these indicators, national health agencies, cancer registries and oncology departments in hospitals would need to start collecting the data required to monitor implementation of the right to health for breast cancer (and may extend those to other cancers). However, in low- and middle- income countries (LMICs) where data availability often presents major challenges, data for these indicators are unlikely to be collected routinely.

Finally, the panel of experts were mostly from Europe. Only three were from North America and none from other world regions. The selection of indicators may therefore have been biased towards Western values or interpretations of what a right-to-health approach means for breast cancer care and management. The approach may have been different if we had involved experts from other regions, especially from LMICs. For this reason, we reiterate that indicators which did not reach a consensus should not be ignored. Some of them may suit specific contexts in LMICs. For instance, indicators 96, 101, 121 and 122 on self-examination may be adapted to countries which do not have the capacity to implement a population-based screening programme for breast cancer or to follow up with treatment of cases diagnosed through the programme [50].

## Conclusions

The right to health is a critical legal tool that may help remedy inequalities in breast cancer survival. However, without indicators to assess how well it is implemented, policymakers and researchers are left with an empty toolbox. We conducted a Delphi study to select indicators of the extent to which the right to health in breast cancer care and management has been implemented. After two rounds of the study, 54 indicators were selected, 3 were rejected, and 97 did not reach consensus.

The 54 indicators selected are consistent with the need to implement and monitor the right-to-health features of a health system that were published by the UN Special Rapporteur on the Right to Health in 2008 [7]. They show how to construct right-to-health indicators to manage a given health condition at the health system level. For breast cancer, key features to implement and monitor include, first, formal recognition of the right to health in breast cancer strategies; then a population-based screening programme; prompt diagnosis; strong referral systems and limited waiting times; the provision of palliative care, attention to the quality of life of survivors and end-of-life care; the availability, accessibility, acceptability and quality (AAAQ) of breast cancer services and medicines; a system of accountability and redress, and the collection of data in a disaggregated form to enable patterns of discrimination to be examined in sufficient detail.

The indicators selected by the panellists suggest that some elements of the right to health may lend themselves better to measurability, such as the AAAQ of health services, facilities and medicines. Some aspects of health systems research may have progressed in the assessment of the right to health over the last 10–15 years, even if these studies are not part of legal scholarship, notably through “people-centred approaches”.

The indicators selected in this Delphi study may guide health policy experts to design national cancer control plans in line with the WHO Global Breast Cancer Initiative. They may also help cancer specialists to implement principles of the right to health in their practice, and to monitor progress. This study adds to the body of literature on monitoring implementation of the right to health with indicators. It offers a set of indicators that can be used to observe the evolution in right-to-health research since the first set of such indicators was published, and it is the first set of indicators to focus on cancer.

## Supplementary Information

**Additional file 1: Appendix 1.** List of indicators, with data sources and rationale for the right to health.

**Additional file 2: Appendix 2.** Glossary of terms and abbreviations.

**Additional file 3: Appendix 3.** List of indicators with their score at the end of the study.

## Data Availability

Not applicable.

## Abbreviations

AAAQ: Availability, Accessibility, Acceptability and Quality of health services, goods and medicines
ASCO: American Society of Clinical Oncology
ESMO: European Society for Medical Oncology
LMICs: Low- and Middle-Income Countries
OECD: Organisation for Economic Co-operation and Development
The Committee: United Nations Committee on Economic, Social and Cultural Rights
The Covenant: International Covenant on Economic, Social and Cultural Rights
WHO: World Health Organisation

## Footnotes

1 We excluded men from this study, although breast cancer does occur in men (about 1% of all breast cancer incidence).

2 “Economic and Social Rights” or “Economic, Social and Cultural Rights” are a category of human rights that protect the conditions needed to live a decent life. Economic and social rights include the right to housing, the right to health, the right to food, the right to education and the right to work. They differ from the other category of human rights that protects fundamental freedoms such as the right to be free from torture or the right to life. This second category is called “Civil and Political Rights.”.

3 The *accessibility* element of the AAAQ framework includes four dimensions: financial, physical, informational and non-discriminatory access. The *acceptability* element is understood as respect for the dignity of patients and the necessity to obtain their consent to treatment and other health interventions.

4 Although there is a growing body of literature and activities on business and human rights principles, including the UN Guiding Principles on Business and Human Rights and a UN Working Group on Business and Human Rights. Information available at: https://www.business-humanrights.org/en/big-issues/un-guiding-principles-on-business-human-rights/ (accessed on 15 July 2022).

5 Indicator 111 was: “Number of treaty-based grounds of discrimination the cancer registry collects data on: sex; ethnic origin, race, or colour; age; disability; language; religion; national origin; socioeconomic status, social status, social origin, or birth; civil status; political status, or political or other opinion; and property; as well as non treaty-based grounds: people living in rural areas, sexual orientation, gender.” It was borrowed from the study on assessment of the right to health and health systems in 194 countries by Backman et al.
