# Supplementary material for: Implementing and monitoring the right to health in breast cancer: selection of indicators using a Delphi process
Source: Int J Equity Health. 2023 Jul 29;22:142. doi: 10.1186/s12939-023-01964-w (PMC10386607; doi:10.1186/s12939-023-01964-w)
Supplement: Supplementary file 1 — Additional file 1: Appendix 1. List of indicators, with data sources and rationale for the right to health. [file 12939_2023_1964_MOESM1_ESM.pdf]

Appendix 1. List of indicators, with data sources and rationale for the right to health

Building blocks and indicators	Data sources	How it contributes to realising the right to health	Principles of the right to health
**1. Health service delivery**			
Structural			
**Indicator #1 (novel)**			
In the National Cancer Plan (NCP) or Non-Communicable Diseases Plan (NCDP) there is a strategy to implement population-based breast cancer screening	National cancer plans, non-communicable diseases plans	Cancer screening allows early detection of breast cancer, which in turn is associated with better chances of survival.	Cost-effectiveness, core obligations
**Indicator #2 (novel)**			
In the NCP or NCDP there a strategy to implement early detection of breast cancer in women at higher risk due to their family history	National cancer plans, non-communicable diseases plans	Women with a family history of breast cancer are more likely to develop breast cancer themselves. International guidelines recommend particular attention to this group such as genetic testing and diagnostic tests. As above, this is to ensure early detection to increase chances of survival.	Cost-effectiveness, core obligations
**Indicator #3 (novel)**			
In the NCP or NCDP there is a strategy to implement an early diagnosis programme in line with *local guidelines*	National cancer plans, non-communicable diseases plans	ESMO and ASCO guidelines are reviewed regularly by experts and constitute an international consensus on the optimal pathway of care. This contributes to the adequacy and quality requirements under the right to health. The indicator was changed to "local guidelines" to be context-specific, whilst still promoting the use of the best standards.	Cost-effectiveness, core obligations, adequacy and quality of health services
**Indicator #4 (novel)**			
In the NCP or NCDP there is a strategy to implement breast cancer national treatment guidelines in line with *local guidelines*	National cancer plans, non-communicable diseases plans	Same as above.	Cost-effectiveness, core obligations, adequacy and quality of health services
**Indicator #5 (novel)**			
In the NCP or NCDP there is a strategy to implement access to breast reconstruction	National cancer plans, non-communicable diseases plans	Losing a breast after a mastectomy can be a traumatic event for a woman. Breast reconstruction allows the woman to recover her breast. This contributes to the acceptability and quality requirements under the right to health.	Acceptability and quality of health services
**Indicator #6 (novel)**			
In the NCP or NCDP there is a strategy to implement access to radiotherapy	National cancer plans, non-communicable diseases plans	Radiotherapy is needed for the treatment of nearly all types of breast cancer. This contributes to the availability of treatment under the right to health.	Availability of treatment
**Indicator #7 (novel)**			
In the NCP or NCDP there is a strategy to implement a multidisciplinary care for breast cancer	National cancer plans, non-communicable diseases plans	ESMO recommends multidisciplinary teams to treat breast cancer as it results in better outcomes. This contributes to the acceptability and quality of services under the right to health.	Acceptability and quality of health services
**Indicator #8 (novel)**			
In the NCP or NCDP there is a strategy to implement palliative/supportive care	National cancer plans, non-communicable diseases plans	Palliative or supportive care is important to improve the quality of life of patients. This contributes to the availability, accessibility, acceptability and quality of services under the right to health.	Availability, accessibility, acceptability and quality of health services
**Indicator #9 (novel)**			
In the NCP or NCDP there is a strategy to implement survivorship care	National cancer plans, non-communicable diseases plans	Survivorship care contributes to the availability, accessibility, acceptability and quality of services under the right to health.	Availability, accessibility, acceptability and quality of health services
**Indicator #10 (novel)**			
In the NCP or NCDP there is a strategy to implement end-of-life care	National cancer plans, non-communicable diseases plans	End-of-life care allows the patients to live in dignity and as pain-free as possible for the last weeks of their life. It contributes to the AAAQ under the right to health.	Availability, accessibility, acceptability and quality of health services
**Indicator #11 (novel)**			
In the NCP or NCDP there is a strategy to implement travel subsidies to access cancer services *for those in need*	National cancer plans, non-communicable diseases plans	This contributes to the financial accessibility of services.	Financial accessibility of health services
**Indicator #12 (novel)**			
In the NCP or NCDP there is a strategy to implement fertility-preservation techniques in younger premenopausal patients	National cancer plans, non-communicable diseases plans	Treatment for breast cancer may decrease fertility. Patients should be informed and offered a choice of treatment to preserve as much as possible fertility. This contributes to the acceptability and quality of treatment.	Acceptability and quality of treatment
Process			
**Indicator #13 (novel)**			
There is a population-based breast cancer screening programme in place	Ministry of Health websites	Cancer screening allows early detection of breast cancer, which in turn is associated with better chances of survival.	Cost-effectiveness, core obligations
**Indicator #14 (novel)**			

Building blocks and indicators	Data sources	How it contributes to realising the right to health	Principles of the right to health
The screening programmes involves annual or biennial mammography in women aged 50-69	Ministry of Health websites	Although there are debates in the scientific community about the optimal age range, the age group reported in this work is determined by international guidelines (ESMO).	Cost-effectiveness, core obligations
**Indicator #15 (novel)**			
The screening programme includes annual MRI and annual mammography, concomitant or alternating, for women with a strong familial history of breast cancer	Ministry of Health websites	This is recommended by international guidelines (ESMO). This contributes to better equity under the right to health by ensuring women with increased risk have chances of getting an early diagnosis. This contributes to the AAAQ of services.	Cost-effectiveness, core obligations, availability, accessibility, acceptability and quality of diagnostic services
**Indicator #16 (novel)**			
The screening programme includes mobile units in hard-to-reach communities	Ministry of Health websites	This contributes to a better geographical reach of the screening programme and gives more accessible screening options to women living away from the main hospitals. This contributes to the non-discriminatory element of the right to health and AAAQ of services.	Cost-effectiveness, core obligations, availability, accessibility, acceptability and quality of diagnostic services, non-discrimination
**Indicator #17 (novel)**			
There is genetic testing in place for women with strong familial history of breast cancer	Ministry of Health websites	This is recommended by international guidelines (ESMO). This contributes to better equity under the right to health by ensuring women with increased risk have chances of getting an early diagnosis. This contributes to the AAAQ of services.	Cost-effectiveness, core obligations, availability, accessibility, acceptability and quality of diagnostic services
**Indicator #18 (novel)**			
There is a referral system in place for women with strong familial history of breast cancer to have a mammogram or MRI	Ministry of Health websites, national health system	If and when breast cancer is suspected, a referral system allows the patient to access diagnostic services early. This contributes to AAAQ of services.	Cost-effectiveness, core obligations, availability, accessibility, acceptability and quality of diagnostic services
**Indicator #19 (novel)**			
There is a referral system in place from primary care to oncology services	Ministry of Health websites, national health system	If and when breast cancer is suspected, a referral system allows the patient to access diagnostic services early. This contributes to AAAQ of services.	Cost-effectiveness, core obligations, availability, accessibility, acceptability and quality of diagnostic services
**Indicator #20 (novel)**			
There is a specified maximum waiting time between diagnostic suspicion in primary care and the first appointment with an oncologist	Ministry of Health websites, national health system	This shows the state's commitment to early diagnosis of breast cancer. The earlier the cancer is diagnosed, the better chances of survival are.	Cost-effectiveness, core obligations
**Indicator #21 (novel)**			
There is a specified maximum waiting time between the confirmed diagnostic and the first appointment for treatment	National guidelines	This shows the state's commitment to early treatment of breast cancer. The earlier the cancer is treated, the better chances of survival are.	Cost-effectiveness, core obligations
**Indicator #22 (novel)**			
The national diagnosis guidelines involve pathological evaluation in line with ESMO or ASCO recommendations	National guidelines	As above. ESMO recommendations: histology from the primary tumour and cytology/histology of the axillary nodes when involvement is suspected.	Cost-effectiveness, core obligations, quality of diagnostic services
**Indicator #23 (novel)**			
The national diagnosis guidelines involve genetic counselling and testing for germline BRCA1 and BRCA2 mutations in high-risk group breast cancer patients	National guidelines	As above, this is in line with international guidelines (ESMO) and allow women with higher risk to be diagnosed early. This contributes to better equity.	Cost-effectiveness, core obligations, quality of diagnostic services and non-discrimination
**Indicator #24 (novel)**			
The national treatment guidelines offer breast reconstruction to all women requiring mastectomy	National guidelines	Losing a breast after a mastectomy can be a traumatic event for a woman. Breast reconstruction allows the woman to recover her breast. This contributes to the acceptability and quality requirements under the right to health.	Acceptability and quality of health services
**Indicator #25 (existing)**			
The number of mammography units, per 1,000,000 inhabitants	OECD statistics health equipment; WHO Global Health Observatory medical equipment (per million women aged 50-69)	This shows the availability of equipment that is necessary for early detection of breast cancer.	Availability of diagnostic equipment
**Indicator #25 disaggregated (novel)**			
Disaggregated by geography	To our knowledge, no availability of data, or case-by-case basis	This shows whether the equipment is evenly spread across the country or whether some regions are left behind. This shows the availability and accessibility requirements under the right to health, as well as non-discrimination based on geography which may be a proxy for socio-economic status.	Availability and accessibility of diagnostic equipment, non-discrimination

Building blocks and indicators	Data sources	How it contributes to realising the right to health	Principles of the right to health
**Indicator #26 (existing)**			
The number of ultrasound machines, per 1,000 inhabitants	Statista for Italy; contact the OECD for international data	This shows the availability of equipment that is necessary for early detection of breast cancer, especially in younger women with denser breast tissue.	Availability of diagnostic equipment
**Indicator #26 disaggregated (novel)**			
Disaggregated by geography	To our knowledge, no availability of data, or case-by-case basis	This shows whether the equipment is evenly spread across the country or whether some regions are left behind. This shows the availability and accessibility requirements under the right to health, as well as non-discrimination based on geography which may be a proxy for socio-economic status.	Availability and accessibility of diagnostic equipment, non-discrimination
**Indicator #27 (existing)**			
The number of radiotherapy units is at least as high as the optimal threshold set by the IAEA (one radiotherapy unit per 500,000 population)	OECD / AGART report (expressed as the number of linear accelerators (LINACs) per million population) referring to the IAEA-DIRAC database 2007-13	This shows the availability of equipment that is necessary for treatment of breast cancer.	Availability of diagnostic equipment
**Indicator #27 disaggregated (novel)**			
Disaggregated by geography	To our knowledge, no availability of data, or case-by-case basis	This shows whether the equipment is evenly spread across the country or whether some regions are left behind. This shows the availability and accessibility requirements under the right to health, as well as non-discrimination based on geography which may be a proxy for socio-economic status.	Availability and accessibility of diagnostic equipment, non-discrimination
**Indicator #28 (novel)**			
Number of disciplines forming part of the breast cancer care unit in a given hospital	Surveys	ESMO recommends multidisciplinary teams to treat breast cancer as it results in better outcomes. This contributes to the acceptability and quality of services under the right to health.	Acceptability and quality of health services
**Indicator #28 disaggregated (novel)**			
Disaggregated by geography	Surveys	Shows potential discrimination based on geography which may be a proxy for socio-economic status.	Non-discrimination
**Indicator #29 (novel)**			
Number of specialists the breast cancer unit collaborates with, out of a psychologist, a social worker, a dietitian, an exercise physiologist, a genetic counsellor, an occupational therapist, a physiotherapist, and a specialist palliative care	Surveys	The disciplines listed in the indicator are recommended by the Australian optimal care pathway for breast cancer. ESMO recommends a physiotherapist, a psychologist and a geneticist. This contributes to the acceptability and quality of services under the right to health.	Acceptability and quality of health services
**Indicator #29 disaggregated (novel)**			
Disaggregated by geography	Surveys	Shows potential discrimination based on geography which may be a proxy for socio-economic status.	Non-discrimination
**Indicator #30 (novel)**			
The breast unit *includes* ASCO recommended integrative therapies to reduce stress, anxiety and depression: music therapy, meditation, stress management, yoga, relaxation, and massage	Surveys	A rights-based approach means that the physical and emotional wellbeing of the patient is taken into account, as opposed to a pure biomedical approach. The efficacy of these therapies were reviewed by the International Society of Oncologists from 1990 to 2015 and further approved by ASCO. They contribute to the AAAQ of services under the right to health and dignity of the person.	Availability, accessibility, acceptability and quality of health services
**Indicator #30 disaggregated (novel)**			
Disaggregated by geography	Surveys	Shows potential discrimination based on geography which may be a proxy for socio-economic status.	Non-discrimination
**Indicator #31 (novel)**			
The breast unit *includes* ASCO recommended integrative therapies to reduce chemotherapy-induced nausea and vomiting: acupuncture and acupressure	Surveys	Same as above.	Availability, accessibility, acceptability and quality of health services
**Indicator #31 disaggregated (novel)**			
Disaggregated by geography	Surveys	Same as above.	Non-discrimination
**Indicator #32 (novel)**			
There is a referral system in place from the breast unit to psychological care	Surveys	A referral system ensures that the patient can access the service in a timely manner while triaging patients to avoid the service to be overloaded. This contributes to the AAAQ of psychological services for breast cancer patients.	Cost-effectiveness and availability, accessibility, acceptability and quality of health services
**Indicator #32 disaggregated (novel)**			
Disaggregated by geography	Surveys	Shows potential discrimination based on geography which may be a proxy for socio-economic status.	Non-discrimination

Building blocks and indicators	Data sources	How it contributes to realising the right to health	Principles of the right to health
**Indicator #33 (novel)**			
There is a referral system in place from the breast unit to reconstructive surgery	Surveys	A referral system ensures that the patient can access the service in a timely manner while triaging patients to avoid the service to be overloaded. This contributes to the AAAQ of reconstructive services for breast cancer patients.	Availability, accessibility, acceptability and quality of health services
**Indicator #33 disaggregated (novel)**			
Disaggregated by geography	Surveys	Shows potential discrimination based on geography which may be a proxy for socio-economic status.	Non-discrimination
**Indicator #34 (novel)**			
There is a trained member of staff acting as patient navigator in the breast unit	Surveys	A trained navigator helps patients navigate between the range of services needed throughout the cancer journey, which may be extremely large and varied. It avoids unnecessary delays and may refrain patients from abandoning their treatment.	Acceptability and quality of health services
**Indicator #34 disaggregated (novel)**			
Disaggregated by geography	Surveys	Shows potential discrimination based on geography which may be a proxy for socio-economic status.	Non-discrimination
Outcome			
**Indicator #35 (novel)**			
Proportion of suspected breast cancer patients with a first consultant appointment within 2 weeks of primary care referral	Primary care data	This shows whether the state's commitment to early diagnosis is applied in practice. The timeline corresponds to the Australian optimal pathway of care for breast cancer. This contributes to the AAAQ of diagnosis for breast cancer.	Cost-effectiveness, core obligations, availability, accessibility, acceptability and quality of health services
**Indicator #35 disaggregated (novel)**			
Disaggregated by wealth quintile and ethnicity	Primary care data, if available	This would show potential discrimination on the basis of socio-economic status and ethnicity.	Non-discrimination
**Indicator #36 (novel)**			
Proportion of breast cancer patients with a surgery appointment within 1 month of a decision to treat with surgery	Secondary care data	This shows whether the state's commitment to early treatment is applied in practice. The timeline corresponds to the Australian optimal pathway of care for breast cancer. This contributes to the AAAQ of diagnosis for breast cancer.	Cost-effectiveness, availability, accessibility, acceptability and quality of health services
**Indicator #36 disaggregated (novel)**			
Disaggregated by wealth quintile and ethnicity	Secondary care data, if available	This would show potential discrimination on the basis of socio-economic status and ethnicity.	Non-discrimination
**Indicator #37 (novel)**			
Proportion of breast cancer patients who started adjuvant systemic therapy within 6 weeks after surgery	Secondary care data	This shows whether the state's commitment to early treatment as per ESMO guidelines is applied in practice. This contributes to the AAAQ of diagnosis for breast cancer.	Cost-effectiveness, core obligations, availability, accessibility, acceptability and quality of health services
**Indicator #37 disaggregated (novel)**			
Disaggregated by wealth quintile and ethnicity	Secondary care data, if available	This would show potential discrimination on the basis of socio-economic status and ethnicity.	Non-discrimination
**Indicator #38 (novel)**			
Proportion of breast cancer patients who started neoadjuvant systemic therapy within 4 weeks after diagnosis	Secondary care data	This shows whether the state's commitment to early treatment as per ESMO guidelines is applied in practice. This contributes to the AAAQ of diagnosis for breast cancer.	Cost-effectiveness, core obligations, availability, accessibility, acceptability and quality of health services
**Indicator #38 disaggregated (novel)**			
Disaggregated by wealth quintile and ethnicity	Secondary care data, if available	This would show potential discrimination on the basis of socio-economic status and ethnicity.	Non-discrimination
**Indicator #39 (existing)**			
Probability of visiting a doctor, adjusted for need	OECD HAG 2019 "use of primary care services"	This would indicate the 4 accessibility elements for primary care under the right to health: physical, financial, information, and non-discriminatory.	Physical, financial, information and non-discriminatory accessibility of primary care services
**Indicator #40 (existing)**			
Proportion of women aged 50-69 who have had a screening mammogram within the last 2 or 3 years	OECD	This shows whether the state's commitment to breast cancer screening is implemented in practice.	Cost-effectiveness, core obligations, availability and accessibility of health services
**Indicator #40 disaggregated (novel)**			

Building blocks and indicators	Data sources	How it contributes to realising the right to health	Principles of the right to health
Disaggregated by wealth quintile and ethnicity	Not yet available at the international level	This would show potential discrimination on the basis of socio-economic status and ethnicity.	Non-discrimination
**Indicator #41 (novel)**			
Proportion of women with strong familial history of breast cancer who have had a mammography or MRI in the last *24 months*	Surveys	This shows whether the state's commitment to breast cancer screening in women at higher risk is implemented in practice.	Cost-effectiveness, core obligations, availability and accessibility of health services
**Indicator #41 disaggregated (novel)**			
Disaggregated by wealth quintile and ethnicity	Surveys	This would show potential discrimination on the basis of socio-economic status and ethnicity.	Non-discrimination
**Indicator #42 (novel)**			
Proportion of women with *advanced stage (IIB-IV)* at diagnosis	Hospital data, or ideally population-based cancer registry data	This shows whether the state's commitment to early diagnosis is implemented in practice.	Cost-effectiveness, core obligations, availability and accessibility of diagnostic services
**Indicator #42 disaggregated (novel)**			
Disaggregated by wealth quintile and ethnicity	Hospital data, or ideally population-based cancer registry data	This would show potential discrimination on the basis of socio-economic status and ethnicity.	Non-discrimination
**Indicator #43 (novel)**			
Proportion of women with advanced breast cancer (stage IV) at diagnosis	Hospital data, or ideally population-based cancer registry data	This shows whether the state's commitment to early diagnosis is implemented in practice.	Cost-effectiveness, core obligations, availability and accessibility of diagnostic services
**Indicator #43 disaggregated (novel)**			
Disaggregated by wealth quintile and ethnicity	Hospital data, or ideally population-based cancer registry data	This would show potential discrimination on the basis of socio-economic status and ethnicity.	Non-discrimination
**Indicator #44 (novel)**			
Proportion of breast cancer patients forgoing or postponing care because of limited availability	National health surveys, potentially European Health Interview Survey	This indicates the availability and physical accessibility of care.	Availability and physical accessibility of care
**Indicator #44 disaggregated (novel)**			
Disaggregated by wealth quintile and ethnicity	Not yet available at the international level	This would show potential discrimination on the basis of socio-economic status and ethnicity.	Non-discrimination
**Indicator #45 (novel)**			
Proportion of breast cancer patients forgoing care because of affordability	National health surveys, potentially European Health Interview Survey	This indicates the financial accessibility of care.	Financial accessibility of care
**Indicator #45 disaggregated (novel)**			
Disaggregated by wealth quintile and ethnicity	Not yet available at the international level	This would show potential discrimination on the basis of socio-economic status and ethnicity.	Non-discrimination
**Indicator #46 (novel)**			
Proportion of women adhering to their treatment	Hospital data, or ideally population-based cancer registry data	This may indicate issues of physical and financial accessibility to treatment.	Physical and financial accessibility of treatment
**Indicator #46 disaggregated (novel)**			
Disaggregated by wealth quintile and ethnicity	Hospital data, or ideally population-based cancer registry data	This would show potential discrimination on the basis of socio-economic status and ethnicity.	Non-discrimination
**Indicator #47 (novel)**			
Proportion of breast cancer patients in high-risk groups who have been offered genetic counselling and testing for germline BRCA1 and BRCA2 mutations	Surveys	This shows whether the state's commitment to detect breast cancer early in high risk women is implemented in practice.	Cost-effectiveness, core obligations, availability and accessibility of quality diagnostic services
**Indicator #47 disaggregated (novel)**			
Disaggregated by wealth quintile and ethnicity	Surveys	This would show potential discrimination on the basis of socio-economic status and ethnicity.	Non-discrimination
**Indicator #48 (novel)**			
Proportion of breast cancer patients who receive *palliative care*	Surveys	This shows whether the state's commitment to provide palliative/supportive care is implemented in practice.	Availability, accessibility, acceptability and quality of health services
**Indicator #48 disaggregated (novel)**			
Disaggregated by wealth quintile and ethnicity	Surveys	This would show potential discrimination on the basis of socio-economic status and ethnicity.	Non-discrimination
**Indicator #49 (novel)**			

Building blocks and indicators	Data sources	How it contributes to realising the right to health	Principles of the right to health
Proportion of women terminally ill with breast cancer who receive end-of-life care	Hospital data	This shows whether the state's commitment to provide end-of-life care is implemented in practice.	Availability, accessibility, acceptability and quality of health services
**Indicator #49 disaggregated (novel)**			
Disaggregated by wealth quintile and ethnicity	Hospital data	This would show potential discrimination on the basis of socio-economic status and ethnicity.	Non-discrimination
**Indicator #50 (novel)**			
Proportion of breast cancer survivors who receive survivorship care	Surveys	This shows whether the state's commitment to provide survivorship care is implemented in practice.	Availability, accessibility, acceptability and quality of health services
**Indicator #50 disaggregated (novel)**			
Disaggregated by wealth quintile and ethnicity	Surveys	This would show potential discrimination on the basis of socio-economic status and ethnicity.	Non-discrimination
**Indicator #51 (existing)**			
Self-reported satisfaction with breast surgery: breast conserving surgery and mastectomy	OECD HAG 2019 PROMs	This indicates acceptability and quality of breast surgery.	Acceptability and quality of breast surgery
**Indicator #51 disaggregated (novel)**			
Disaggregated by wealth quintile and ethnicity	Not yet available at the international level	This would show potential discrimination on the basis of socio-economic status and ethnicity.	Non-discrimination
**Indicator #52 (novel)**			
Proportion of women who were offered breast reconstruction immediately after their mastectomy, excluding inflammatory breast cancer cases	Surveys	This indicates the AAAQ of breast surgery and shows whether patients are treated with dignity. The recommendation is based on ESMO guidelines.	Availability, accessibility, acceptability and quality of health services
**Indicator #52 disaggregated (novel)**			
Disaggregated by wealth quintile and ethnicity	Surveys	This would show potential discrimination on the basis of socio-economic status and ethnicity.	Non-discrimination
**Indicator #53 (novel)**			
Self-reported satisfaction of breast cancer patients with their diagnosis journey: mammogram, ultrasound and breast biopsies	Surveys	This indicates the AAAQ of diagnosis for breast cancer, especially the acceptability and quality of services.	Availability, accessibility, acceptability and quality of health services
**Indicator #53 disaggregated (novel)**			
Disaggregated by wealth quintile and ethnicity	Surveys	This would show potential discrimination on the basis of socio-economic status and ethnicity.	Non-discrimination
**Indicator #54 (novel)**			
Self-reported satisfaction of breast cancer patients with their treatment	Surveys	This indicates the AAAQ of treatment for breast cancer, especially the acceptability and quality of services.	Availability, accessibility, acceptability and quality of treatment
**Indicator #54 disaggregated (novel)**			
Disaggregated by wealth quintile and ethnicity	Surveys	This would show potential discrimination on the basis of socio-economic status and ethnicity.	Non-discrimination
**Indicator #55 (novel)**			
Self-reported satisfaction of breast cancer patients with the palliative/supportive care received throughout their cancer journey	Surveys	This indicates the AAAQ of palliative/supportive care for breast cancer, especially the acceptability and quality of services.	Availability, accessibility, acceptability and quality of treatment
**Indicator #55 disaggregated (novel)**			
Disaggregated by wealth quintile and ethnicity	Surveys	This would show potential discrimination on the basis of socio-economic status and ethnicity.	Non-discrimination
**Indicator #56 (novel)**			
Proportion of breast cancer patients who were followed by one or more of the following during their treatment: a psychologist, a social worker, a dietitian, an exercise physiologist, a genetic counsellor, an occupational therapist, a physiotherapist, and a specialist palliative care.	Hospital data or Surveys	This shows whether the state's commitment to treat breast cancer with multidisciplinary teams and to provide integrated care is applied in practice.	Acceptability and quality of health services
**Indicator #56 disaggregated (novel)**			
Disaggregated by wealth quintile and ethnicity	Hospital data or Surveys	This would show potential discrimination on the basis of socio-economic status and ethnicity.	Non-discrimination
**Indicator #57 (novel)**			
Proportion of breast cancer patients who received integrative therapies to help manage stress, anxiety and depression such as music therapy, meditation, stress management, yoga, relaxation, and massage	Hospital data or Surveys	This shows whether the state's commitment to provide integrated care is applied in practice.	Acceptability and quality of health services
**Indicator #57 disaggregated (novel)**			

Building blocks and indicators	Data sources	How it contributes to realising the right to health	Principles of the right to health
Disaggregated by wealth quintile and ethnicity	Hospital data or Surveys	This would show potential discrimination on the basis of socio-economic status and ethnicity.	Non-discrimination
**Indicator #58 (novel)**			
Proportion of breast cancer patients who received integrative therapies to help reduce chemotherapy-induced nausea and vomiting such as acupuncture and acupressure	Hospital data or Surveys	This shows whether the state's commitment to provide integrated care is applied in practice.	Acceptability and quality of health services
**Indicator #58 disaggregated (novel)**			
Disaggregated by wealth quintile and ethnicity	Hospital data or Surveys	This would show potential discrimination on the basis of socio-economic status and ethnicity.	Non-discrimination
**2. Health financing**			
Structural			
**Indicator #59 (novel)**			
The NCP or NCDP addresses costs of implementation of the breast cancer strategy	National cancer plans, non-communicable diseases plans	Shows the financial commitment of the state and fulfils the obligation of a national strategy under the right to health.	Core obligations
**Indicator #60 (existing)**			
Total government health expenditure per capita	OECD HAG 2019, expressed in USD and adjusted for purchasing power parity	Shows the state's commitment as a duty bearer of the right to health.	Cost-effectiveness, maximum available resources, financial accessibility of health services
**Indicator #61 (existing)**			
Share of government spending and out-of-pocket payment out of the total spending on health per capita	OECD HAG 2019, expressed in USD and adjusted for purchasing power parity	Shows the state's commitment as a duty bearer of the right to health.	Cost-effectiveness, maximum available resources, financial accessibility of health services
**Indicator #62 (novel)**			
Total government spending on health compared with military as a percentage of GDP	Government's websites for public spending, expressed as the coefficient = military spending / health spending. Health spending taken from OECD HAG.	Shows the state's commitment as a duty bearer of the right to health. Indicates whether the state has used its maximum available resources, an obligation under the right to health.	Maximum available resources
**Indicator #63 (existing)**			
Proportion of total health expenditure dedicated to cancer	OECD cancer care study 2013 (https://read.oecd-ilibrary.org/social-issues-migration-health/cancer-care_9789264181052-en#page21)	Shows the state's commitment as a duty bearer of the right to health.	Cost-effectiveness, maximum available resources, financial accessibility of cancer services
**Indicator #64 (existing)**			
The state has a *social health insurance system*	OECD HAG 2019	Shows the state's commitment as a primary duty bearer of the right to health. Indicates the financial accessibility of health services and care.	Cost-effectiveness, financial accessibility of health services
Process			
**Indicator #65 (novel)**			
The *social health insurance system* covers diagnostic services for breast cancer (i.e., biopsy, mammogram and ultrasound)	Ministry of Health websites, national health system	Indicates the financial accessibility of diagnostic services.	Financial accessibility of diagnostic services
**Indicator #66 (novel)**			
The *social health insurance system* covers breast cancer treatment (i.e., hormone therapy and chemotherapy)	Ministry of Health websites, national health system	Indicates the financial accessibility of treatment.	Financial accessibility of treatment
**Indicator #67 (novel)**			
The *social health insurance system* covers radiotherapy for breast cancer	Ministry of Health websites, national health system	Indicates the financial accessibility of treatment.	Financial accessibility of treatment
**Indicator #68 (novel)**			
The *social health insurance system* covers palliative care for breast cancer	Ministry of Health websites, national health system	Indicates the financial accessibility of palliative care.	Financial accessibility of palliative care
Outcome			
**Indicator #69 (novel)**			
Proportion of breast cancer patients who benefit from the *social health insurance system*	National surveys of breast cancer patients	Measures the population covered by universal health coverage.	Financial accessibility of health services and treatment
**Indicator #69 disaggregated (novel)**			
Disaggregated by wealth quintile, ethnicity *and migration status*	National surveys of breast cancer patients	This would show potential discrimination on the basis of socio-economic status and ethnicity.	Non-discrimination
**Indicator #70 (novel)**			
Proportion of costs covered for breast cancer care by the *social health insurance system*	Computing of the proportion of expenditure for breast cancer care covered under government schemes or compulsory health insurance	Measures the costs covered by universal health coverage.	Cost-effectiveness, maximum available resources, financial accessibility of care
**Indicator #71 (novel)**			
Proportion of breast cancer patients with catastrophic health expenditure	National surveys of breast cancer patients	Measures financial accessibility of health services and care.	Financial accessibility of health services and care

Building blocks and indicators	Data sources	How it contributes to realising the right to health	Principles of the right to health
**Indicator #71 disaggregated (novel)**			
Disaggregated by wealth quintile and ethnicity	National surveys of breast cancer patients	This would show potential discrimination on the basis of socio-economic status and ethnicity.	Non-discrimination
**3. Medicines**			
Structural			
**Indicator #72 (existing)**			
The Constitution, Bill of Rights or other statute recognises the right to access essential medicines	WHO report 2008 https://www.who.int/medicines/areas/human_rights/Perehudoff_report_constitutions_2008.pdf	Indicates the formal recognition of the right to health and access to essential medicines.	Legal recognition
**Indicator #73 (existing)**			
There is an official national medicines policy to provide access to essential medicines	WHO, E-DRUG, Lancet Commission report (2016)	Indicates the state's commitment to access to essential medicines.	Core obligations
**Indicator #74 (novel)**			
The NCP or NCDP mentions breast cancer medicines included in the WHO Essential Medicines List	WHO's Essential Medicines and Health Products Information Portal, national cancer plans, non-communicable diseases plans	Indicates the state's commitment to access to essential medicines for breast cancer.	Availability of medicines
Process			
**Indicator #75 (existing)**			
Coverage status of medicines for metastatic breast cancer *that are included in the WHO Essential Medicines List*: not approved, approved but not covered, approved and covered	OECD report 2020 tables AC1 and AC6 if relevant https://www.oecd.org/health/health-systems/Addressing-Challenges-in-Access-to-Oncology-Medicines-Analytical-Report.pdf	Shows the availability of essential medicines for breast cancer.	Availability of medicines
**Indicator #76 (novel)**			
Proportion of breast cancer medicines *included in the WHO Essential Medicines List* that are available in the country and covered by public funding	National surveys of states for the pharmaceutical market	Shows the financial accessibility of essential medicines for breast cancer.	Financial accessibility of medicines
**Indicator #77 (novel)**			
Proportion of generics in the pharmaceutical market for breast cancer medicines	National surveys of states for the pharmaceutical market	Shows the financial accessibility of essential medicines for breast cancer.	Financial accessibility of medicines
Outcome			
**Indicator #78 (novel)**			
Proportion of hospitals with breast cancer medicines shortage	Surveys	Indicates the actual availability of medicines for breast cancer.	Availability of medicines
**Indicator #78 disaggregated (novel)**			
Disaggregated by geography	Surveys	This would show geographical disparities, which may be a proxy for socio-economic status.	Non-discrimination
**Indicator #79 (novel)**			
Proportion of hospitals with palliative medicines shortage	Surveys	Indicates the actual availability of palliative medicines.	Availability of palliative medicines
**Indicator #79 disaggregated (novel)**			
Disaggregated by geography	Surveys	This would show geographical disparities, which may be a proxy for socio-economic status.	Non-discrimination
**4. Health workforce**			
Structural			
**Indicator #80 (existing)**			
The state has a national health workforce strategy	WHO database: www.who.int/countries/en/	Indicates the state's commitment to the availability of a health workforce, which is an obligation under para43(f) of General Comment 14 (to adopt and implement a national public health strategy).	Core obligations
**Indicator #81 (novel)**			
The state law includes provision for adequate remuneration of doctors *and* nurses, *including* oncologists	WHO Global Atlas of the Health Workforce; LABORSTA; CISDOC–ILO; ILO NATLEX; World Medical Association; British Medical Association; British Medical Journal; and DFID	Indicates the state's commitment to quality and acceptability of the health workforce.	Acceptability and quality of health workforce
**Indicator #82 (novel)**			
The state's workforce policies or programmes provide incentives to promote stationing in rural areas of doctors, nurses and oncologists	WHO Global Atlas of the Health Workforce; LABORSTA; CISDOC–ILO; ILO NATLEX; World Medical Association; British Medical Association; British Medical Journal; and DFID	Indicates the state's commitment to the availability and accessibility of health services and care.	Availability and accessibility of health services, non-discrimination
**Indicator #83 (novel)**			
Human rights are a compulsory part of the national curriculum for the training of doctors, nurses and oncologists	World Medical Association; British Medical Association; Medical Students International Network; World Association of School as an Instrument for Peace; Human Rights Education Network; International Federation of Medical Students' Associations; Centre for International Health and Development	Indicates the state's commitment to provide appropriate training for health personnel, which is an obligation under para 44(e) of General Comment 14 (to provide appropriate training for health personnel, including education on health and human rights).	Core obligations
Process			

Building blocks and indicators	Data sources	How it contributes to realising the right to health	Principles of the right to health
**Indicator #84 (existing)**			
Median time spent for a new consultation, as reported by oncologists	Seruga et al (need to enquire for raw data)	May indicate the acceptability and quality of consultations with oncologists.	Acceptability and quality of health services
**Indicator #85 (existing)**			
Median job satisfaction score of oncologists	Seruga et al (need to enquire for raw data)	May indicate whether oncologists work under adequate conditions.	Acceptability and quality of health services
Outcome			
**Indicator #86 (existing)**			
Prevalence of certified oncologists per 1,000,000 population	WHO Cancer Country Profiles, expressed in number of qualified oncologists per million population / OECD data on cancer care	Indicates the actual availability of oncology services.	Availability of health services
**Indicator #86 disaggregated (novel)**			
Disaggregated by geography	Not yet available at the international level	This would show geographical disparities, which may be a proxy for socio-economic status.	Non-discrimination
**5. Health information**			
Structural			
**Indicator #87 (novel)**			
The state law requires protection of the confidentiality of personal health data	Government's website and GDPR tracker (requires a subscription): https://uk.practicallaw.thomsonreuters.com/w-013-1949?transitionType=Default&contextData=(sc.Default)&firstPage=true	Indicates the state's commitment to acceptability of health services by upholding confidentiality of personal sensitive data.	Acceptability of health services
**Indicator #88 (novel)**			
The state law allows for *controlled* exceptions to the restrictions of sharing personal health data for epidemiological research *by non-commercial organisations*	Government's website and GDPR tracker (requires a subscription): https://uk.practicallaw.thomsonreuters.com/w-013-1949?transitionType=Default&contextData=(sc.Default)&firstPage=true	Indicates the state's commitment to epidemiological research, which is an obligation under para 43(f) of General Comment 14 (to adopt and implement a national public health strategy and plan of action, on the basis of epidemiological evidence, addressing the health concerns of the whole population).	Core obligations
**Indicator #89 (novel)**			
The state law requires cancer registration	IARC's cancer incidence in five continents	Indicates the state's commitment to availability of health data for cancer to inform epidemiological research and monitor the effect of health policies.	Core obligations
**Indicator #90 (existing)**			
The state law requires informed consent to treatment and other health interventions	WHO genomics website in the first instance: http://www.who.int/genomics/public/patientrights/en/index.html or International Health Digest: http://www.who.int/idhl-rils/frame.cfm?language=english	Indicates the state's commitment to acceptability of health services by upholding the right to informed consent for treatment and other health interventions.	Acceptability of health services
**Indicator #91 (novel)**			
The NCP or NCDP *plans for* cancer research, *independent from pharmaceutical companies*	National cancer plans, non-communicable diseases plans	Indicates the state's commitment to epidemiological research, which is an obligation under para 43(f) of General Comment 14 (to adopt and implement a national public health strategy and plan of action, on the basis of epidemiological evidence, addressing the health concerns of the whole population).	Core obligations
**Indicator #92 (novel)**			
The NCP or NCDP protects the right to seek and receive health information	National cancer plans, non-communicable diseases plans	Indicates the state's commitment to protect the right to health information and the commitment to accessibility of health services by providing access to information.	Accessibility (information) of health services, core obligations
**Indicator #93 (novel)**			
The NCP or NCDP addresses communication of information throughout the pathway of care for breast cancer, from screening through to referral, diagnosis, treatment options and palliative care	National cancer plans, non-communicable diseases plans	Indicates the state's commitment to access health information. Indicator based on ESMO recommendation. This indicator may be in the form of a score, the number corresponding to the items being covered by the communication of information (screening, referral, diagnosis, treatment, palliative care).	Informational accessibility of health services
**Indicator #94 (novel)**			
The NCP or NCDP addresses the needs of patients from culturally and linguistically diverse backgrounds	National cancer plans, non-communicable diseases plans	Indicates the state's commitment to non-discriminatory access to health care and services.	Acceptability of heath services, non-discrimination
**Indicator #95 (novel)**			
The NCP or NCDP addresses participation of patients in decisions that affect them	National cancer plans, non-communicable diseases plans	Indicates the state's commitment to participation of patients, which is a requirement under the right to health.	Participation
**Indicator #96 (novel)**			

Building blocks and indicators	Data sources	How it contributes to realising the right to health	Principles of the right to health
The NCP or NCDP addresses awareness of breast self-examination	National cancer plans, non-communicable diseases plans	Indicates the state's commitment to cost-effectiveness of health policies, which is a requirement under para 44(c) of General Comment 14 (to take measures to prevent, treat and control epidemic and endemic diseases) and (d) (to provide education and access to information concerning the main health problems in the community, including methods of preventing and controlling them).	Cost-effectiveness, accessibility (information) of diagnostic services, core obligations
**Indicator #97 (novel)**			
There is a charter of breast cancer patients' rights freely available and accessible to everyone	National cancer patients associations and Europa Donna: https://www.europadonna.org/	Indicates the state's commitment to the right to health information, which is an obligation under para 44(d) of General Comment 14 (see above.)	Accessibility (information) of health services, core obligations
Process			
**Indicator #98 (novel)**			
There is a national campaign to <u>*raise awareness about*</u> the screening programme	Ministry of Health websites	Shows availability of information about the screening programme.	Accessibility (information) of health services, core obligations
**Indicator #99 (novel)**			
The screening programme is implemented through community outreach activities	Ministry of Health websites, breast cancer patient associations	Shows availability and accessibility of information about the screening programme.	Accessibility (information) of health services, core obligations
**Indicator #100 (novel)**			
The screening programme includes information on the potential disadvantages and benefits of mammographic screening	Ministry of Health websites	Shows the availability and quality of information about the screening programme.	Accessibility (information) of health services, core obligations
**Indicator #101 (novel)**			
There is a national strategy to raise awareness on breast self-examination	Ministry of Health websites	Shows availability of information about breast self-examination, which is a key and cost-effective practice to detect early suspicions of breast cancer.	Accessibility (information) of health services
**Indicator #102 (novel)**			
There is a national breast cancer patients association <u>*independent from the pharmaceutical industry*</u>	Europa Donna database, Ministry of Health links to patients associations	Shows the availability of patient support, including information delivered in an accessible way.	Accessibility (information) of health services
**Indicator #103 (novel)**			
The personnel in the breast unit are trained to communicate information on diagnosis and treatment options, including side effects and survival, repeatedly, verbally and in writing, in a comprehensive and easily understandable form to patients	Surveys	Shows the accessibility and quality of information. This indicator is worded as per the ESMO guidelines.	Accessibility (information) and quality of health services
**Indicator #104 (novel)**			
The personnel in the breast unit presents all options available to the patient beside a mastectomy, and explains in an easily understandable way psychological distress that may be associated with a mastectomy	Surveys	Shows the accessibility, acceptability and quality of information delivered to the patient so that she can make informed choices regarding their mastectomy.	Accessibility, acceptability and quality of health information, core obligations
**Indicator #105 (novel)**			
The personnel in the breast unit discusses breast reconstruction techniques individually taking into account anatomic, treatment- and patient-related factors and preferences	Surveys	Shows the availability, accessibility, acceptability and quality of information around breast reconstruction. Indicators worded as per ESMO guidelines.	Accessibility, acceptability and quality of health information, core obligations
**Indicator #106 (novel)**			
The breast unit website or other official source of information includes fertility issues and fertility-preservation techniques	Internet search on a sample of hospitals	Shows the availability, accessibility, acceptability and quality of information around fertility issues due to chemotherapy. Indicators worded as per ESMO guidelines.	Accessibility, acceptability and quality of health information, core obligations
**Indicator #107 (novel)**			
The breast cancer unit uses trained interpreters when communicating with patients from culturally and linguistically diverse background	Surveys	Shows the availability, accessibility, acceptability and quality of communication of information to breast cancer patients.	Accessibility, acceptability and quality of health information, core obligations
**Indicator #108 (novel)**			
The breast unit has a participatory process in place to include patients into decisions that affect them individually	Surveys	Shows the availability of a participatory mechanism for breast cancer patients.	Participation
**Indicator #109 (novel)**			
The breast unit directs patients to reliable, patient-centred websites	Surveys	Shows the availability, accessibility, acceptability and quality of information delivered to breast cancer patients.	Accessibility, acceptability and quality of health information, core obligations
**Indicator #110 (novel)**			
There is a state funded cancer registry	Online search and governments' websites	Shows the availability of health data to inform epidemiological research and monitor the effect of health policies.	Availability of epidemiological data, core obligations
**Indicator #111 (novel)**			

Building blocks and indicators	Data sources	How it contributes to realising the right to health	Principles of the right to health
Number of treaty-based grounds of discrimination the cancer registry collects data on: sex; ethnic origin, race, or colour; age; disability; language; religion; national origin; socioeconomic status, social status, social origin, or birth; civil status; political status, or political or other opinion; and property; as well as non treaty-based grounds: people living in rural areas, sexual orientation, gender	VENUSCANCER questionnaires for some of these grounds, enquiries to cancer registries	Shows the availability of health data that may allow to conduct studies on discrimination.	Non-discrimination
Outcome			
**Indicator #112 (novel)**			
Proportion of women aged 50-69 who are aware of the importance of breast cancer screening	Surveys	Indicates the actual access to information and awareness on breast cancer.	Accessibility of health information
**Indicator #112 disaggregated (novel)**			
Disaggregated by wealth quintile and ethnicity	Surveys	This would show potential discrimination on the basis of socio-economic status and ethnicity.	Non-discrimination
**Indicator #113 (novel)**			
Proportion of women who had their mammography screening in the last *24 months* and were informed of the potential disadvantages and benefits of mammographic screening	Surveys	Indicates the actual access to information and awareness on the advantages and disadvantages of screening.	Accessibility of health information
**Indicator #113 disaggregated (novel)**			
Disaggregated by wealth quintile and ethnicity	Surveys	This would show potential discrimination on the basis of socio-economic status and ethnicity.	Non-discrimination
**Indicator #114 (novel)**			
Proportion of breast cancer patients who feel they have received sufficient, comprehensive and easily understandable information, including on treatment side effects and survival, to be involved in decisions about their care	Surveys	Indicates the acceptability and quality of information received.	Acceptability and quality of health information
**Indicator #114 disaggregated (novel)**			
Disaggregated by wealth quintile, ethnicity, *and language*	Surveys	This would show potential discrimination on the basis of socio-economic status and ethnicity.	Non-discrimination
**Indicator #115 (novel)**			
Proportion of women from culturally or linguistically diverse backgrounds who were offered an interpreter to communicate during their breast cancer journey	Surveys	Indicates the acceptability and quality of information received.	Acceptability and quality of health information
**Indicator #115 disaggregated (novel)**			
Disaggregated by wealth quintile and ethnicity	Surveys	This would show potential discrimination on the basis of socio-economic status and ethnicity.	Non-discrimination
**Indicator #116 (novel)**			
Proportion of breast cancer patients who feel they have been involved in decisions about their care	Surveys	Indicates the participatory level of breast cancer patients in decisions that affect them.	Participation
**Indicator #116 disaggregated (novel)**			
Disaggregated by wealth quintile and ethnicity	Surveys	This would show potential discrimination on the basis of socio-economic status and ethnicity.	Non-discrimination
**Indicator #117 (novel)**			
Proportion of breast cancer patients who feel they have received sufficient, comprehensive and easily understandable information about their mastectomy	Surveys	Indicates the acceptability and quality of information received.	Acceptability and quality of health information
**Indicator #117 disaggregated (novel)**			
Disaggregated by wealth quintile and ethnicity	Surveys	This would show potential discrimination on the basis of socio-economic status and ethnicity.	Non-discrimination
**Indicator #118 (novel)**			
Proportion of breast cancer patients who feel they have been involved in making the decision to undergo a mastectomy	Surveys	Indicates the acceptability and quality of information received.	Acceptability and quality of health information
**Indicator #118 disaggregated (novel)**			
Disaggregated by wealth quintile and ethnicity	Surveys	This would show potential discrimination on the basis of socio-economic status and ethnicity.	Non-discrimination
**Indicator #119 (novel)**			
Proportion of premenopausal patients who received sufficient, comprehensive and easily understandable information about fertility issues and fertility-preservation techniques before receiving their treatment	Surveys	Indicates the acceptability and quality of information received. This type of information is recommended by ESMO.	Acceptability and quality of health information
**Indicator #119 disaggregated (novel)**			
Disaggregated by wealth quintile and ethnicity	Surveys	This would show potential discrimination on the basis of socio-economic status and ethnicity.	Non-discrimination
**Indicator #120 (novel)**			

Building blocks and indicators	Data sources	How it contributes to realising the right to health	Principles of the right to health
Proportion of women with breast cancer who felt that they received sufficient information at all stages of their cancer journey, from screening through to referral, diagnosis, treatment options and palliative care	Surveys	Indicates the acceptability and quality of information received.	Acceptability and quality of health information
**Indicator #120 disaggregated (novel)**			
Disaggregated by wealth quintile and ethnicity	Surveys	This would show potential discrimination on the basis of socio-economic status and ethnicity.	Non-discrimination
**Indicator #121 (novel)**			
Proportion of women who are aware of the importance of breast self-examination	Surveys	Indicates the actual awareness of breast self-examination, which is a key cost-effective practice to detect early suspicions of breast cancer. This is in line with paras 44 (c) and (d) of General Comment 14 (to take measures to prevent, treat and control epidemic and endemic diseases; and to provide education and access to information concerning the main health problems in the community, including methods of preventing and controlling them).	Accessibility of health information
**Indicator #121 disaggregated (novel)**			
Disaggregated by wealth quintile and ethnicity	Surveys	This would show potential discrimination on the basis of socio-economic status and ethnicity.	Non-discrimination
**Indicator #122 (novel)**			
Proportion of women regularly undertaking breast self-examination	Surveys	Indicates whether the cost-effective practice is performed in practice. If not, further investigation will be needed to find out why. There may be cultural barriers.	Cost-effectiveness, accessibility of health information
**Indicator #122 disaggregated (novel)**			
Disaggregated by wealth quintile and ethnicity	Surveys	This may indicate potential cultural barriers to this practice, or discrimination on the access to information.	Non-discrimination
**Indicator #123 (novel)**			
Self-reported reason for women diagnosed with locally advanced or advanced breast cancer for not seeking care at an earlier stage of the disease	Surveys	Indicates the barriers to seeking care.	Accessibility of health care
**Indicator #123 disaggregated (novel)**			
Disaggregated by wealth quintile and ethnicity	Surveys	This would show potential discrimination on the basis of socio-economic status and ethnicity.	Non-discrimination
**6. Governance / Leadership**			
Structural			
**Indicator #124 (existing)**			
The Constitution, Bill of Rights, or other statute recognises the right to health	WHO report 2008 https://www.who.int/medicines/areas/human_rights/Perehudoff_report_constitutions_2008.pdf	Indicates whether the state is committed to the right to health with a national legal recognition.	Legal recognition
**Indicator #125 (novel)**			
The state has transparent rules on lobbying, including a public lobbying register	Global Public Procurement Database: https://www.globalpublicprocurementdata.org/gppd/	Rules on lobbying would address the commercial determinants of health and limit the power of powerful multinationals to increase health risks (tobacco, soft drinks, alcohol). This is in line with the state's duty to protect the right to health against private actors.	Responsibility to protect
**Indicator #126 (novel)**			
The state has an NCP or NCDP addressing breast cancer specifically	WHO Cancer Country Profiles	Indicates the state's commitment to realise the right to health, paragraphs 43(f) (to adopt and implement a national public health strategy and plan of action) and 44(c) of General Comment 14 (to take measures to prevent, treat and control epidemic and endemic diseases).	Core obligations
**Indicator #127 (novel)**			
The NCP or NCDP addresses accountability of the state and health institutions	National cancer plans, non-communicable diseases plans	Accountability is a key principle of the right to health. Without accountability, the right holders do not have the tool to enforce their rights and there is no opportunity for lessons learned and improvement.	Accountability
**Indicator #128 (novel)**			
The NCP or NCDP addresses vulnerable populations such as ethnic minorities, refugees, homosexuals, and LBGQ+	National cancer plans, non-communicable diseases plans	Vulnerable groups are key to the right to health. They must not be left behind and their needs must be specifically addressed as part of the non-discriminatory obligation of the right to health.	Non-discrimination
**Indicator #129 (novel)**			
The NCP or NCDP explicitly recognises the right to health	National cancer plans, non-communicable diseases plans	Indicates the state's commitment to the right to health in relation to cancer care and control specifically.	Legal recognition
**Indicator #130 (novel)**			

Building blocks and indicators	Data sources	How it contributes to realising the right to health	Principles of the right to health
The NCP or NCDP includes a protection against discrimination	National cancer plans, non-communicable diseases plans	Indicates the state's commitment against discrimination in relation to cancer care and control specifically.	Non-discrimination
**Indicator #131 (novel)**			
The NCP or NCDP includes an explicit commitment to universal access to cancer services and treatment	National cancer plans, non-communicable diseases plans	This indicates the state's core obligation of the right to health listed at paragraph 43(a) of General Comment 14 (to ensure the right of access to health facilities, goods and services on a non-discriminatory basis, especially for vulnerable or marginalized groups).	Non-discrimination, core obligations
**Indicator #132 (novel)**			
The NCP or NCDP includes a set of targets and progress indicators *specific to breast cancer*	National cancer plans, non-communicable diseases plans	Targets and indicators are necessary to measure progress towards the realisation of the right to health. This is a key tool for the "progressive realisation" element of the right to health. Monitoring is also a core obligation listed at paragraph 43(f) of General Comment 14 (the strategy and plan of action shall be devised, and periodically reviewed, on the basis of a participatory and transparent process; they shall include methods, such as right to health indicators and benchmarks, by which progress can be closely monitored).	Core obligations
**Indicator #133 (novel)**			
The state undertook a health impact assessment before adopting the national cancer plan	National cancer plans, non-communicable diseases plans	This indicates the state's core obligation of the right to health listed at paragraph 43(f) of General Comment 14 (to adopt and implement a national public health strategy and plan of action, on the basis of epidemiological evidence, addressing the health concerns of the whole population).	Core obligations
**Indicator #134 (novel)**			
The health impact assessment explicitly included the right to health	National cancer plans, non-communicable diseases plans	Indicates the state's commitment to realise the right to health.	Legal recognition
**Indicator #135 (novel)**			
The NCP or NCDP includes a requirement for the participation of marginalised groups	National cancer plans, non-communicable diseases plans	Participation of affected groups is a key principle of the right to health. It is also necessary to realise the core obligation listed at paragraph 43(a) of General Comment 14 (to ensure the right of access to health facilities, goods and services on a non-discriminatory basis, especially for vulnerable or marginalized groups) and paragraph 43 (f) (the process by which the strategy and plan of action are devised, as well as their content, shall give particular attention to all vulnerable or marginalized groups).	Participation, non-discrimination
**Indicator #136 (novel)**			
The Ministry of Health collaborates with the Ministries of Employment, Education, or Finance on cancer policy	National cancer plans, non-communicable diseases plans	Collaboration and health in all policies or a "one health" approach is a key component of the right to health.	Efficiency, collaboration
Process			
**No indicator**			
Outcome			
**Indicator #137 (existing)**			
State's corruption index	World Bank Governance Indicators	A state's level of corruption may indicate poor rule of law and a less democratic society, which are necessary prerequisites for the realisation of the right to health.	Cost-effectiveness, maximum available resources
**Indicator #138 (existing)**			
State's rule of law index	World Bank Governance Indicators	Same as above.	Cost-effectiveness, maximum available resources
**7. Underlying determinants of breast cancer**			
Structural			
**Indicator #139 (novel)**			
The state is a party to the Framework Convention on Tobacco Control	UN treaty collection: https://treaties.un.org/pages/ViewDetails.aspx?src=TREATY&mtdsg_no=IX-4&chapter=9&clang=_en	Indicates the state's commitment to the right to health and addresses the underlying determinants of health (tobacco control). The underlying determinants of health are a key component to address for the realisation of the right to health (General Comment 14, General Comment 22, Special Rapporteur on the right to health reports).	Prevention (core obligation)
**Indicator #140 (existing)**			

Building blocks and indicators	Data sources	How it contributes to realising the right to health	Principles of the right to health
The state has an operational policy, strategy or action plan to reduce the burden of tobacco use	Government's websites	Indicates the state's commitment to the right to health, and commitment to tackle the underlying determinants of health. The state also has a core obligation to adopt a national plan or strategy to address the health concerns of the population (para 43(f) of General Comment 14) and to prevent, treat and control endemic and epidemic diseases (paragraph 44(c)).	Prevention (core obligation)
**Indicator #141 (existing)**			
The state has an operational policy, strategy or action plan to reduce overweight and obesity	Government's websites	Same as above.	Prevention (core obligation)
**Indicator #142 (existing)**			
The state has an operational policy, strategy or action plan to reduce physical inactivity and/or promote physical activity	Government's websites	Same as above.	Prevention (core obligation)
**Indicator #143 (existing)**			
The state has an operational policy, strategy or action plan to reduce the harmful use of alcohol	Government's websites	Same as above.	Prevention (core obligation)
Process			
**No indicator**			
Outcome			
**Indicator #144 (novel)**			
The country air pollution is below the WHO recommended annual mean (10 µg/m3 for fine particulate matter PM2.5 and 20 µg/m3 for coarse particulate matter PM10)	Global Health Observatory: https://www.who.int/data/gho/data/indicators/indicator-details/GHO/concentrations-of-fine-particulate-matter-(pm2-5)	Indicates a key outcome on the underlying determinants of health.	Prevention (core obligation)
**Indicator #145 (existing)**			
Proportion of adult women who are smokers	Expressed as a percentage of all adult women. World Bank: https://data.Worldbank.org/indicator/SH.PRV.SMOK.FE	Same as above.	Prevention (core obligation)
**Indicator #146 (existing)**			
Proportion of adult women who are overweight and obese	Expressed as a percentage of all adult women. Global Obesity Observatory: https://data.worldobesity.org/tables/prevalence-of-adult-overweight-obesity-2/	Same as above.	Prevention (core obligation)
**Indicator #147 (existing)**			
Prevalence of hazardous drinking among women	OECD: https://www.oecd-ilibrary.org/content/paper/5js1qwkz2p9s-en?crawler=true	Same as above.	Prevention (core obligation)
**8. Redress**			
Structural			
**Indicator #148 (existing)**			
There is an accessible pre-judicial mechanism to lodge complaints alleging breach of obligations connected to the right to health	Google search	Indicates the state's commitment to provide redress for victims, to enforce the right to health, and to ensure accountability of wrongdoers and health institutions.	Accountability
Process			
**Indicator #149 (novel)**			
Number of court cases litigated on cancer in a given year	Global Health and Human Rights database (category is NCDs, not cancer specifically): https://www.globalhealthrights.org/	Indicates both the level of access to justice and the issues in the realisation of the right to health for cancer patients specifically. The caveat is that only a few wealthy individuals may have access to litigation in practice, so the court cases would not be representative of poorer or marginalised individuals.	Accountability
**Indicator #150 (novel)**			
Proportion of class actions among the cases litigated on cancer in a given year	Manual search in Global Health and Human Rights database	Indicates the level of access to justice and systemic issues in the realisation of the right to health for cancer patients. Class actions may be more representative of the population and systemic issues as it is brought by a group of affected individuals, as opposed to one person. The outcome of the litigation may also be fairer at a group level, rather than creating inequalities between those who could access litigation and those who could not in individual cases.	Accountability
**Indicator #151 (novel)**			
The breast unit has a formal complaints mechanism for patients	Surveys	Indicates the access to justice for breast cancer patients at the hospital level. This may be more meaningful and accessible for patients than litigation in court.	Accountability
**Indicator #151 disaggregated (novel)**			
Disaggregated by geography	Surveys	Shows potential discrimination based on geography which may be a proxy for socio-economic status.	Non-discrimination
Outcome			
**No indicator**			

Building blocks and indicators	Data sources	How it contributes to realising the right to health	Principles of the right to health
**ADDITIONAL INDICATORS SUGGESTED IN ROUND 1, FOR SELECTION IN ROUND 2**			
**Indicator #152 (novel) - to replace indicator #13**			
Proportion of the population at risk participating in the screening programme	National health statistics	Cancer screening allows early detection of breast cancer, which in turn is associated with better chances of survival.	Cost-effectiveness, core obligations
**Indicator #153 (novel)**			
Prevalence of certified nurses per 1,000,000 population	OECD data	Indicates availability of health personnel.	Availability of health workforce
**Indicator #154 (existing)**			
The State has ratified key human rights treaties recognising the right to health	OHCHR website	Indicates whether the state is committed to the right to health as protected by international human rights law.	Legal recognition

End of document

Words in italic and underlined show reformulation as per the panel's suggestions.
