# Supplementary material for: Implementing and monitoring the right to health in breast cancer: selection of indicators using a Delphi process
Source: Int J Equity Health. 2023 Jul 29;22:142. doi: 10.1186/s12939-023-01964-w (PMC10386607; doi:10.1186/s12939-023-01964-w)
Supplement: Supplementary file 2 — Additional file 2: Appendix 2. Glossary of terms and abbreviations. [file 12939_2023_1964_MOESM2_ESM.pdf]

Appendix 2. Glossary of terms and abbreviations

Term	Definition	Reference
AAAQ	All health services, goods and facilities shall be available, accessible, acceptable and of good quality (AAAQ). The precise nature of these elements will depend on the conditions prevailing in a particular State.<br>Available: functioning public health and health-care facilities, goods and services, as well as programmes, have to be available in sufficient quantity within the State party. The precise nature will vary depending on numerous factors, including the State party's development level.<br>Accessible: health facilities, goods and services have to be accessible to everyone without discrimination, within the jurisdiction of the State party. It includes: non-discrimination; physical accessibility; affordability and information accessibility.<br>Acceptable: all health facilities, goods and services must be respectful of medical ethics and culturally appropriate as well as being designed to respect confidentiality and improve the health status of those concerned.<br>Good quality: health facilities must be scientifically and medically appropriate and of good quality. This requires skilled medical personnel, scientifically approved and unexpired drugs and hospital equipment, safe and potable, and adequate sanitation.	Appendix 1 Backman et al (2008) The Lancet
Accountability	Accountability: the right to health brings with it the crucial requirement of accessible, transparent and effective mechanisms of monitoring and accountability. Those with right-to-health responsibilities must be held to account in relation to the discharge of their duties, with a view to identifying successes and difficulties; so far as necessary, policy and other adjustments can then be made. Examples of accountability mechanisms are:<br>1.Judicial, e.g. judicial review of executive acts and omissions<br>2.Quasi-judicial, e.g. NHRIs (see below), human rights treaty-bodies<br>3.Administrative, e.g. human rights impact assessment<br>4.Political, e.g. parliamentary committees<br>5.Social, e.g. civil society movements<br>The accountability mechanism should exist at the national, regional (if available) and international levels. Rights-holders are also entitled to effective remedies when duty-bearers have failed to discharge their right to health obligations. These remedies may take the form of restitution, rehabilitation, compensation, satisfaction or guarantees of non-repetition.	Appendix 1 Backman et al (2008) The Lancet
Advanced breast cancer	Breast cancer classed as stage IV on the Tumour Nodes Metastases staging system (TNM). Stage IV means that the tumour has spread to other areas of the body.	ESMO guide for breast cancer patients

Term	Definition	Reference
Age-standardised five-year net survival from breast cancer	Five-year net survival is the cumulative probability that cancer patients survive their cancer for at least 5 years, after controlling for the risks of death from other causes. Net survival is expressed as a percentage. Cancer survival estimates<br>are age-standardised with the International Cancer Survival Standard (ICSS) weights.	CONCORD programme, OECD HAG
Air pollution	Particulate matter (PM) is a common proxy indicator for air pollution. It affects more people than any other pollutant. It consists of a complex mixture of solid and liquid particles of organic and inorganic substances suspended in the air. While particles with a diameter of 10 microns or less, (≤ PM10) can penetrate and lodge deep inside the lungs, the even more health-damaging particles are those with a diameter of 2.5 microns or less, (≤ PM2.5). PM2.5 can penetrate the lung barrier and enter the blood system. Air quality measurements are typically reported in terms of daily or annual mean concentrations of PM10 particles per cubic meter of air volume (m3). Routine air quality measurements typically describe such PM concentrations in terms of micrograms per cubic meter (μg/m3). When sufficiently sensitive measurement tools are available, concentrations of fine particles (PM2.5 or smaller), are also reported.	WHO factsheet on ambient air pollution
Biopsy	A medical procedure in which a small sample of cells or tissue is taken for examination under a microscope.	ESMO guide for breast cancer patients
BRCA mutations	Expression used to refer to the BRCA1 or BRCA2 genes which, when mutated (not functioning properly), are associated with a very high risk of breast and ovarian cancers.	ESMO guide for breast cancer patients
Breast cancer	Breast cancer is a type of cancer that starts in the breast. Cancer starts when cells begin to grow out of control. Breast cancer cells usually form a tumour that can often be seen on an x-ray or felt as a lump. Breast cancer occurs almost entirely in women, but men can get breast cancer, too.	American Cancer Society
Breast cancer care	Includes all services, medical devices, goods and medicines needed to screen for, diagnose, and treat breast cancer.	My definition
Breast cancer patients	For the purpose of this piece of research, includes all women diagnosed with breast cancer stage I to IV in the general population, and excludes men diagnosed with breast cancer.	My definition
Breast conserving surgery	Surgery to remove a tumour and the surrounding breast tissue while retaining as much of the breast as possible.	ESMO guide for breast cancer patients
Cancer journey	Experience of the entire care continuum, from seeking care through to screening, diagnosis, surgery, treatment, palliative and end-of-life care.	My definition

Term	Definition	Reference
Catastrophic health expenditure	Out of pocket payments that exceed a predefined percentage of the resources available to a household to pay for health<br>care. Data provided by the OECD define these resources as household consumption minus a standard amount representing basic spending on food, rent and utilities (water, electricity, gas and other fuels). The threshold used to define households with catastrophic spending in the literature varies between 5% and 40%. Both the OECD and WHO use the 40% threshold.	OECD Health at a Glance 2019
Chemotherapy	A type of cancer treatment using medicine that kills the cancer cells by damaging them, so that they cannot reproduce and spread.	ESMO guide for breast cancer patients
Disaggregation	Typically data are reported for whole populations or as aggregated data. Aggregated data do not always represent the true status of health of populations. Human rights require that, so far as practical, all relevant data are disaggregated on the prohibited grounds of discrimination. This helps in monitoring the situation of marginal groups, such as women living in poverty, indigenous people, minorities and so on.	Appendix 1 Backman et al (2008) The Lancet
Early diagnosis programme	Identifies and addresses barriers to diagnostic and treatment services in the population and among service providers. It builds service capacity and quality and establishes referral pathways.	WHO Europe
End-of-life care	For patients with incurable cancer, end-of-life case primarily focusses on making the patient comfortable and providing adequate relief of physical and psychological symptoms. For example, palliative sedation to induce unconsciousness can relieve intolerable pain, dyspnoea, delirium or convulsions.	ESMO guide for breast cancer patients
Ethnicity / race	Categories used to differentiate groups in the general population according to their ethnic or racial origin. For the purpose of this piece of research, race and ethnicity are used interchangeably and data analysed will use the categories pre-defined by each state. We acknowledge the limit that these categories may differ between states, therefore limiting data comparability. As recommended by the OECD, states should develop national diversity statistical standards to allow linking data across sources, and ensure the representation of hard-to-reach populations such as indigenous communities.	OECD, Diversity statistics in the OECD, How do OECD countries collect data on ethnic, racial and indigenous identity? (2018)
General Comment 14	Adopted by the Committee on Economic, Social and Cultural Rights (CESCR) in May 2000, provides an authoritative interpretation of contours and contents of the right to the highest attainable standard of health. CESCR is the body which monitors the International Covenant on Economic, Social and Cultural Rights and also publishes its interpretation of the provisions of the Covenant in the form of general comments.	Appendix 1 Backman et al (2008) The Lancet
Generic	A pharmaceutical product which has the same qualitative and quantitative composition in active substances and the same pharmaceutical form as the reference product, and whose bioequivalence with the reference product has been demonstrated. Generics may be either branded (generics with a specific trade name) or unbranded (identified using the international non-proprietary name and the name of the company).	OECD Health at a Glance 2019

Term	Definition	Reference
Genetic counselling	Process through which knowledge about the genetic aspects of illnesses is shared by trained professionals with those who are at an increased risk of either having a heritable disorder or of passing it on to their unborn offspring.	WHO definition
Government health expenditure per capita	This includes spending by government-based programmes, such as social insurance, but excludes out-of-pocket spending. Includes expenditure on medical services and goods, population health and prevention programmes, as well as administration of the health system.	OECD Health at a Glance 2019
Health workers	A generic term to include all those developing, delivering, monitoring and evaluating preventive, curative and rehabilitative health 'plans' in the private and public health sector. It also includes traditional healers whether or not they have been incorporated into the health system. Pursuant to the obligation to protect, the state has an obligation to ensure that traditional healers are aware of and carry out their responsibilities regarding the right to health.	Appendix 1 Backman et al (2008) The Lancet
Hormone or endocrine therapy	A type of anticancer therapy that reduces the supply of hormones to hormone receptor-dependent breast cancers.	ESMO guide for breast cancer patients
Human rights-based approach to h	A human rights-based approach to health specifically aims at realizing the right to health and other health-related human rights. Health policy making and programming are to be guided by human rights standards and principles and aim at developing capacity of duty bearers to meet their obligations and empowering rights-holders to effectively claim their health rights. Elimination of all forms of discrimination is at the core of a human rights-based approach to health. It comprises the AAAQ framework (availability, accessibility, acceptability and quality of health services, goods and medicines) and the active and informed participation of affected groups; accountability of duty-bearers; and non-discrimination. The state must fulfil its obligations to respect, protect and fulfil.<br>Respect: not to interfere directly or indirectly with the enjoyment of the right to health, e.g. refrain from limiting access to health-car services or marketing unsafe drugs.<br>Protect: prevent third parties from interfering with the right to health, e.g. ensure that private companies provide safe environmental conditions for their employees and surrounding communities.<br>Fulfil: adopt appropriate legislative, administrative, budgetary, judicial, promotional and other measures to fully realise the right to health.	WHO factsheet on a human rights-based approach to health (2009)
Integrated care	Integrated health services delivery is defined as an approach to strengthen people-centred health systems through the promotion of the comprehensive delivery of quality services across the life-course, designed according to the multidimensional needs of the population and the individual and delivered by a coordinated multidisciplinary team of providers working across settings and levels of care. It should be effectively managed to ensure optimal outcomes and the appropriate use of resources based on the best available evidence, with feedback loops to continuously improve performance and to tackle upstream causes of ill health and to promote well-being through intersectoral and multisectoral actions.	WHO Regional Office for Europe
International guidelines	Refer to the latest guidelines on breast cancer diagnosis or treatment published by ESMO or ASCO.	My definition

Term	Definition	Reference
Locally advanced breast cancer	Breast cancer classed as stage IIB to III on the Tumour Nodes Metastases staging system (TNM). The stage depends on how big the tumour is (T), whether the cancer has spread to the lymph nodes (N), and whether the cancer has spread to distant sites, or metastases (M).	ESMO guide for breast cancer patients
Mammography	An x-ray of the breasts that can detect early breast cancers. The number of mammography units is measured by the OECD per 1,000,000 inhabitants.	ESMO guide for breast cancer patients
Mastectomy	Surgery to remove a breast.	ESMO guide for breast cancer patients
Medicines	Active pharmaceutical ingredients, diagnostic tools, vaccines, biopharmaceuticals and other healthcare technologies.	Appendix 1 Backman et al (2008) The Lancet
National cancer plan	A National Cancer Plan (NCP) is a public health programme designed to reduce the number of people diagnosed with cancer each year, the level of survival and the number of deaths from cancer, and to improve the quality of life of cancer patients, through the systematic and equitable implementation of evidence-based strategies for: prevention, early detection, diagnosis, treatment, rehabilitation, palliation and research for innovative solutions and evaluation of outcomes. It is designed with the aim of making the best use of available resources.<br>We do not insist on the word ‘plan’. A State might use a different word e.g. strategy or policy (e.g. General Comment 14, paragraph 43, uses a different wording; it refers to “a national health policy with detailed plan”). The plan could be a set of separate documents, provided they form one reasonably cohesive package. The plan must be reflective of the national and local situation and incorporates both the public and private sector.	European Partnership Action Against Cancer and Appendix 1 Backman et al (2008) The Lancet
Non-communicable diseases	Non-communicable diseases, also known as chronic diseases, tend to be of long duration and are the result of a combination of genetic, physiological, environmental and behavioural factors. The main types of non-communicable diseases are cardiovascular diseases (such as heart attacks and strokes), cancers, chronic respiratory diseases (such as chronic obstructive pulmonary disease and asthma) and diabetes.	WHO factsheet on non-communicable diseases
Non-discrimination	The principle of non-discrimination seeks to guarantee that human rights are exercised without discrimination of any kind based on race, colour, sex, language, religion, political or other opinion, national or social origin, property, birth or other status such as disability, age, marital and family status, sexual orientation and gender identity, health status, place of residence, economic and social situation.	Committee on Economic, Social and Cultural Rights, General Comment No. 20, Non-discrimination in economic, social and cultural rights; 2009.
Oncology services	Include all services needed to diagnose and treat cancer, including radiology, pathology, surgery, and oncology.	My definition

Term	Definition	Reference
Out of pocket payments	Expenditures borne directly by a patient where neither public nor private insurance cover the full cost of the health good or service. They include cost-sharing and other expenditure paid directly by private households and should also ideally include estimations of informal payments to health providers.	OECD Health at a Glance 2019
Palliative / supportive care	Palliative care is an approach that improves the quality of life of patients and their families who are facing problems associated with life-threatening illness. It prevents and relieves suffering through the early identification, correct assessment and treatment of pain and other problems, whether physical, psychosocial or spiritual. A state may use the term "supportive care" instead, which we understand as palliative care.	WHO factsheet on palliative care
Participation	Active and informed participation of individuals and communities in decision-making that has a bearing on their health.	Appendix 1 Backman et al (2008) The Lancet
Policy	The term may be use interchangeably with "plan" or "strategy". The plan could be a set of separate documents, provided they form a cohesive and related package, or it could be one document. The important point is that, whatever it is called and whatever form it is in, it is reflective of the national and local situation and incorporates both the public and private sector.	Appendix 1 Backman et al (2008) The Lancet
Population-based breast cancer screening programme	Breast cancer screening involves the systematic use of testing through mammography in an asymptomatic population to detect and treat breast cancer at an earlier stage than if the woman were diagnosed when symptomatic, or non-malignant growths. "Population-based" means that the entire targeted population, typically women aged 50 to 69, is invited for screening. The programme should include both initial invitations and re-calls.	Based on WHO position paper on mammography screening (2014)
Primary care	Addresses the main health problems in the community, providing promotive, preventive, curative and rehabilitative services accordingly. It should be sustained by integrated, functional and mutually supportive referral systems, leading to the progressive improvement of comprehensive health care for all, and giving priority to those most in need.	Alma-Ata Declaration (1978)
Radiotherapy	Radiotherapy is one of the most widely used therapies for cancer treatment. It consists of using radiation in its different forms (X-rays, Gamma rays, particles) to harm and destroy tumours, either alone or in combination with surgery or chemotherapy. The availability of equipment is expressed by the IAEA as the number of linear accelerators (LINACs) per million population.	IAEA
Regular mammogram	Annual or biennial, as recommended by the latest ESMO clinical practice guidelines for diagnosis, treatment and follow-up of early breast cancer.	My definition

Term	Definition	Reference
Right to health / right to the highest attainable standard of health / right of everyone to the enjoyment of the highest attainable standard of physical and mental health	The right to health is a fundamental human right, including freedoms and entitlements. It does not mean 'a right to be healthy'; the government cannot fully ensure good health as it is influenced by factors which are in whole or in part outside the State's control, such as individual susceptibility to ill-health and adoption of unhealthy lifestyles. However, the entitlements include the right to an effective and integrated health system, encompassing health care and the underlying determinants of health, e.g. access to education, safe water, sanitation and food, which is responsive to national and local priorities, and accessible, available, acceptable and of good quality to all. Participation by the population in all health-related decision-making at the community, national and international levels is a component of the right to health. The right to the highest attainable standard of health is codified in numerous legally binding international and regional human rights treaties. The right is also enshrined in numerous national constitutions. The right to health, as with all human rights, is linked to other rights, such as right to life and freedom from discrimination.<br>States have duties to respect, protect and fulfil the right to the highest attainable standard of health. These duties are equally applicable to medical care and the underlying determinants of health. The obligation to respect requires States to refrain from interfering directly or indirectly with the enjoyment of the right to health. The obligation to protect requires States to take measures that prevent third parties from interfering with the right to health. Finally, the obligation to fulfil requires States to adopt appropriate legislative, administrative, budgetary, judicial, promotional and other measures towards the full realisation of the right to health.	Appendix 1 Backman et al (2008) The Lancet
Social health insurance system	Social Health Insurance (SHI) is a form of financing and managing health care based on risk pooling. SHI pools both the health risks of the people on one hand, and the contributions of individuals, households, enterprises, and the government on the other. Thus, it protects people against financial and health burden and is a relatively fair method of financing health care.	WHO report (2003)
Strategy	The term may be use interchangeably with "plan" or "policy". The plan could be a set of separate documents, provided they form a cohesive and related package, or it could be one document. The important point is that, whatever it is called and whatever form it is in, it is reflective of the national and local situation and incorporates both the public and private sector.	Appendix 1 Backman et al (2008) The Lancet
Strong familial history of breast ca	A woman has a strong familial history of breast cancer when a first-degree relative (defined as a mother, sibling or child) was also diagnosed with breast cancer, with or without proven BRCA mutations.	ESMO clinical practice guidelines for early breast cancer
Surveys	A survey is an investigation about the characteristics of a given population by means of collecting data from a sample of that population and estimating their characteristics through the systematic use of statistical methodology.	OECD glossary of statistical terms

Term	Definition	Reference
Survivorship	Survivorship focuses on health and the physical, psychological, social and economic issues affecting people after the end of the primary treatment for cancer. Post treatment cancer survivors range from people having no disease after finishing treatment, people who continue to receive treatment to reduce the risk of the cancer coming back and people with well controlled disease and few symptoms, who receive treatment to manage cancer as a chronic disease. Survivorship care includes issues related to follow-up care, the management of late side-effects of treatment, the improvement of quality of life and psychological and emotional health. Survivorship care includes also future anticancer treatment where applicable. Family members, friends and caregivers should also be considered as part of the survivorship experience.	ESMO patient guide on survivorship
Treatment	Includes surgery, hormone therapy, chemotherapy, and radiotherapy.	My definition
Ultrasound scan	A type of medical scan where sound waves are converted into images by a computer. It is used to detect breast cancer that may not be visible with a mammography, especially in young women with denser breast tissue.	ESMO guide for breast cancer patients
Underlying determinants of health	Include access to safe and potable water and adequate sanitation, an adequate supply of safe food, nutrition and housing, healthy occupational and environmental conditions, and access to health-related education and information, including on sexual and reproductive health. Other determinants of health include gender, poverty and social exclusion.	Appendix 1 Backman et al (2008) The Lancet
Wealth quintile	Division of the general population in five equal groups according to the distribution of wealth, i.e. from the richest 20% to the poorest 20% of the population.	My definition

References

Allemani C, Matsuda T, Di Carlo V, et al. Global surveillance of trends in cancer survival 2000-14 (CONCORD-3) *The Lancet* 2018; **391**: 1023-75.

American Cancer Society. What is breast cancer? Available at: https://www.cancer.org/cancer/breast-cancer/about/what-is-breast-cancer.html

Backman G, et al. Health systems and the right to health: an assessment of 194 countries. The Lancet 2008; 372: 2047-85.

Balestra, C. Fleischer, L. Diversity statistics in the OECD. Working Paper No. 96 (2018).

Cardoso F, Kyriakides S, Ohno S, et al. Early breast cancer: ESMO Clinical Practice Guidelines. Annals of oncology 2019; 30: 1194-220.

Declaration of Alma-Ata. International Conference on Primary Health Care, Alma-Ata, USSR, 6-12 September 1978.

European Partnership for Action Against Cancer, National Cancer Plans. Available at: http://www.epaac.eu/national-cancer-plans

European Society for Medical Oncology. Breast cancer: a guide for patients (2018).

European Society for Medical Oncology. Patient guide on survivorship (2017).

International Atomic Energy Agency. Cancer treatment: Radiotherapy. Available at: https://www.iaea.org/topics/cancer-treatment-radiotherapy

International Labour Organization. International Labour Standards on Social security. Available at: https://www.ilo.org/global/standards/subjects-covered-by-international-labour-standards/social-security/lang--en/index.htm

Organisation for Economic Co-operation and Development. Health at a Glance (2019).

Organisation for Economic Co-Operation and Development. Glossary of statistical terms. Available at: https://stats.oecd.org/glossary/

Screening programmes: a short guide. Increase effectiveness, maximize benefits and minimize harm. Copenhagen: WHO Regional Office for Europe; 2020.

UN Committee on Economic, Social and Cultural Rights, General comment No. 20. (2009) E/C.12/GC/20.

WHO Regional Office for Europe. Integrated care models: an overview (2016).

WHO, Office of the High Commissioner for Human Rights, A human rights-based approach to health. Factsheet (2009).

World Health Organization. Ambient (outdoor) air pollution. Factsheet (2018).

World Health Organization. Noncommunicable diseases. Factsheet (2018).

World Health Organization. Palliative care. Factsheet (2020).

World Health Organization. WHO position paper on mammography screening (2014).

World Health Organization. Social Health Insurance: Report of a Regional Expert Group Meeting. New Delhi, India (2003).

Abbreviation	Full quote
AAAQ	The human rights framework of Availability, Accessibility, Acceptability and Quality of health services, goods and medicines
ASCO	American Society of Clinical Oncology
ESMO	European Society for Medical Oncology
HAG	Health at a Glance, OECD biennial publication on healthcare quality indicators
HRBA	Human Rights-Based Approach to Health
IAEA	International Atomic Energy Agency
ILO	International Labour Organisation
NCDP	Non-communicable diseases plan
NCP	National cancer plan
OECD	Organisation for Economic Co-operation and Development
WHO	World Health Organisation
