# Supplementary material for: Implementing and monitoring the right to health in breast cancer: selection of indicators using a Delphi process
Source: Int J Equity Health. 2023 Jul 29;22:142. doi: 10.1186/s12939-023-01964-w (PMC10386607; doi:10.1186/s12939-023-01964-w)
Supplement: Supplementary file 3 — Additional file 3: Appendix 3. List of indicators with their score at the end of the study. [file 12939_2023_1964_MOESM3_ESM.pdf]

Appendix 3: list of indicators with their score at the end of the study

Building blocks and indicators	ROUND1			ROUND 2			Selected?
	Reject	Indifferent	Select	Reject	Indifferent	Select	
**1. Health service delivery**							
Structural							
**Indicator #1 (novel)**							
In the National Cancer Plan (NCP) or Non-Communicable Diseases Plan (NCDP) there is a strategy to implement population-based breast cancer screening	18%	9%	73%	0%	13%	88%	yes
**Indicator #2 (novel)**							
In the NCP or NCDP there a strategy to implement early detection of breast cancer in women at higher risk due to their family history	27%	0%	73%	13%	13%	75%	no
**Indicator #3 (novel)**							
In the NCP or NCDP there is a strategy to implement an early diagnosis programme in line with *local guidelines*	27%	9%	64%	11%	22%	67%	no
**Indicator #4 (novel)**							
In the NCP or NCDP there is a strategy to implement breast cancer national treatment guidelines in line with *local guidelines*	27%	9%	64%	11%	22%	67%	no
**Indicator #5 (novel)**							
In the NCP or NCDP there is a strategy to implement access to breast reconstruction	36%	0%	64%	56%	0%	44%	no
**Indicator #6 (novel)**							
In the NCP or NCDP there is a strategy to implement access to radiotherapy	9%	0%	91%	Selected in round 1			yes
**Indicator #7 (novel)**							
In the NCP or NCDP there is a strategy to implement a multidisciplinary care for breast cancer	36%	0%	64%	33%	0%	67%	no

Building blocks and indicators	ROUND1			ROUND 2			Selected?
	Reject	Indifferent	Select	Reject	Indifferent	Select	
**Indicator #8 (novel)**							
In the NCP or NCDP there is a strategy to implement palliative/supportive care	9%	0%	91%	Selected in round 1			yes
**Indicator #9 (novel)**							
In the NCP or NCDP there is a strategy to implement survivorship care	27%	0%	73%	0%	0%	100%	yes
**Indicator #10 (novel)**							
In the NCP or NCDP there is a strategy to implement end-of-life care	9%	0%	91%	Selected in round 1			yes
**Indicator #11 (novel)**							
In the NCP or NCDP there is a strategy to implement travel subsidies to access cancer services *for those in need*	36%	0%	64%	44%	11%	44%	no
**Indicator #12 (novel)**							
In the NCP or NCDP there is a strategy to implement fertility-preservation techniques in younger premenopausal patients	36%	18%	45%	33%	22%	44%	no
Process							
**Indicator #13 (novel)**							
There is a population-based breast cancer screening programme in place	18%	9%	73%	13%	13%	75%	no
**Indicator #14 (novel)**							
The screening programmes involves annual or biennial mammography in women aged 50-69	55%	18%	27%	63%	25%	13%	no
**Indicator #15 (novel)**							
The screening programme includes annual MRI and annual mammography, concomitant or alternating, for women with a strong familial history of breast cancer	45%	0%	55%	44%	0%	56%	no
**Indicator #16 (novel)**							

Building blocks and indicators	ROUND1			ROUND 2			Selected?
	Reject	Indifferent	Select	Reject	Indifferent	Select	
The screening programme includes mobile units in hard-to-reach communities	40%	0%	60%	44%	0%	56%	no
**Indicator #17 (novel)**							
There is genetic testing in place for women with strong familial history of breast cancer	36%	0%	64%	25%	25%	50%	no
**Indicator #18 (novel)**							
There is a referral system in place for women with strong familial history of breast cancer to have a mammogram or MRI	27%	0%	73%	25%	0%	75%	no
**Indicator #19 (novel)**							
There is a referral system in place from primary care to oncology services	9%	0%	91%	Selected in round 1			yes
**Indicator #20 (novel)**							
There is a specified maximum waiting time between diagnostic suspicion in primary care and the first appointment with an oncologist	9%	9%	82%	Selected in round 1			yes
**Indicator #21 (novel)**							
There is a specified maximum waiting time between the confirmed diagnostic and the first appointment for treatment	9%	9%	82%	Selected in round 1			yes
**Indicator #22 (novel)**							
The national diagnosis guidelines involve pathological evaluation in line with ESMO or ASCO recommendations	27%	0%	73%	0%	13%	88%	yes
**Indicator #23 (novel)**							
The national diagnosis guidelines involve genetic counselling and testing for germline BRCA1 and BRCA2 mutations in high-risk group breast cancer patients	27%	9%	64%	13%	13%	75%	no

Building blocks and indicators	ROUND1			ROUND 2			Selected?
	Reject	Indifferent	Select	Reject	Indifferent	Select	
**Indicator #24 (novel)**							
The national treatment guidelines offer breast reconstruction to all women requiring mastectomy	36%	0%	64%	11%	33%	56%	no
**Indicator #25 (existing)**							
The number of mammography units, per 1,000,000 inhabitants	27%	18%	55%	22%	22%	56%	no
**Indicator #25 disaggregated (novel)**							
Disaggregated by geography	36%	0%	64%	33%	0%	67%	no
**Indicator #26 (existing)**							
The number of ultrasound machines, per 1,000 inhabitants	27%	9%	64%	25%	0%	75%	no
**Indicator #26 disaggregated (novel)**							
Disaggregated by geography	36%	0%	64%	25%	0%	75%	no
**Indicator #27 (existing)**							
The number of radiotherapy units is at least as high as the optimal threshold set by the IAEA (one radiotherapy unit per 500,000 population)	18%	9%	73%	13%	0%	88%	yes
**Indicator #27 disaggregated (novel)**							
Disaggregated by geography	27%	0%	73%	13%	0%	88%	yes
**Indicator #28 (novel)**							

Building blocks and indicators	ROUND1			ROUND 2			Selected?
	Reject	Indifferent	Select	Reject	Indifferent	Select	
Number of disciplines forming part of the breast cancer care unit in a given hospital	36%	18%	45%	78%	0%	22%	no
**Indicator #28 disaggregated (novel)**							
Disaggregated by geography	40%	10%	50%	71%	0%	29%	no
**Indicator #29 (novel)**							
Number of specialists the breast cancer unit collaborates with, out of a psychologist, a social worker, a dietitian, an exercise physiologist, a genetic counsellor, an occupational therapist, a physiotherapist, and a specialist palliative care	40%	10%	50%	67%	11%	22%	no
**Indicator #29 disaggregated (novel)**							
Disaggregated by geography	50%	0%	50%	63%	13%	25%	no
**Indicator #30 (novel)**							
The breast unit *includes* ASCO recommended integrative therapies to reduce stress, anxiety and depression: music therapy, meditation, stress management, yoga, relaxation, and massage	55%	0%	45%	67%	0%	33%	no
**Indicator #30 disaggregated (novel)**							
Disaggregated by geography	45%	0%	55%	63%	0%	38%	no
**Indicator #31 (novel)**							
The breast unit *includes* ASCO recommended integrative therapies to reduce chemotherapy-induced nausea and vomiting: acupuncture and acupressure	45%	9%	45%	44%	11%	44%	no
**Indicator #31 disaggregated (novel)**							
Disaggregated by geography	55%	0%	45%	50%	0%	50%	no
**Indicator #32 (novel)**							
There is a referral system in place from the breast unit to psychological care	9%	0%	91%	Selected in round 1			yes

Building blocks and indicators	ROUND1			ROUND 2			Selected?
	Reject	Indifferent	Select	Reject	Indifferent	Select	
**Indicator #32 disaggregated (novel)**							
Disaggregated by geography	9%	0%	91%	Selected in round 1			yes
**Indicator #33 (novel)**							
There is a referral system in place from the breast unit to reconstructive surgery	36%	0%	64%	13%	13%	75%	no
**Indicator #33 disaggregated (novel)**							
Disaggregated by geography	36%	0%	64%	13%	13%	75%	no
**Indicator #34 (novel)**							
There is a trained member of staff acting as patient navigator in the breast unit	9%	9%	82%	Selected in round 1			yes
**Indicator #34 disaggregated (novel)**							
Disaggregated by geography	18%	0%	82%	Selected in round 1			yes
Outcome							
**Indicator #35 (novel)**							
Proportion of suspected breast cancer patients with a first consultant appointment within 2 weeks of primary care referral	9%	9%	82%	Selected in round 1			yes
**Indicator #35 disaggregated (novel)**							
Disaggregated by wealth quintile and ethnicity	18%	9%	73%	22%	11%	67%	no
**Indicator #36 (novel)**							
Proportion of breast cancer patients with a surgery appointment within 1 month of a decision to treat with surgery	18%	9%	73%	0%	25%	75%	no
**Indicator #36 disaggregated (novel)**							

Building blocks and indicators	ROUND1			ROUND 2			Selected?
	Reject	Indifferent	Select	Reject	Indifferent	Select	
Disaggregated by wealth quintile and ethnicity	27%	9%	64%	13%	25%	63%	no
**Indicator #37 (novel)**							
Proportion of breast cancer patients who started adjuvant systemic therapy within 6 weeks after surgery	27%	9%	64%	13%	13%	75%	no
**Indicator #37 disaggregated (novel)**							
Disaggregated by wealth quintile and ethnicity	27%	9%	64%	13%	13%	75%	no
**Indicator #38 (novel)**							
Proportion of breast cancer patients who started neoadjuvant systemic therapy within 4 weeks after diagnosis	27%	9%	64%	13%	13%	75%	no
**Indicator #38 disaggregated (novel)**							
Disaggregated by wealth quintile and ethnicity	27%	9%	64%	13%	13%	75%	no
**Indicator #39 (existing)**							
Probability of visiting a doctor, adjusted for need	40%	10%	50%	44%	22%	33%	no
**Indicator #40 (existing)**							
Proportion of women aged 50-69 who have had a screening mammogram within the last 2 or 3 years	36%	18%	45%	33%	11%	56%	no
**Indicator #40 disaggregated (novel)**							
Disaggregated by wealth quintile and ethnicity	36%	18%	45%	25%	13%	63%	no
**Indicator #41 (novel)**							
Proportion of women with strong familial history of breast cancer who have had a mammography or MRI in the last *24 months*	36%	9%	55%	33%	0%	67%	no
**Indicator #41 disaggregated (novel)**							

Building blocks and indicators	ROUND1			ROUND 2			Selected?
	Reject	Indifferent	Select	Reject	Indifferent	Select	
Disaggregated by wealth quintile and ethnicity	45%	9%	45%	25%	13%	63%	no
**Indicator #42 (novel)**							
Proportion of women with *advanced stage (IIB-IV)* at diagnosis	27%	9%	64%	22%	0%	78%	no
**Indicator #42 disaggregated (novel)**							
Disaggregated by wealth quintile and ethnicity	27%	9%	64%	13%	0%	88%	yes
**Indicator #43 (novel)**							
Proportion of women with advanced breast cancer (stage IV) at diagnosis	18%	9%	73%	0%	0%	100%	yes
**Indicator #43 disaggregated (novel)**							
Disaggregated by wealth quintile and ethnicity	10%	10%	80%	0%	0%	100%	yes
**Indicator #44 (novel)**							
Proportion of breast cancer patients forgoing or postponing care because of limited availability	9%	9%	82%	Selected in round 1			yes
**Indicator #44 disaggregated (novel)**							
Disaggregated by wealth quintile and ethnicity	9%	9%	82%	Selected in round 1			yes
**Indicator #45 (novel)**							
Proportion of breast cancer patients forgoing care because of affordability	9%	9%	82%	Selected in round 1			yes
**Indicator #45 disaggregated (novel)**							
Disaggregated by wealth quintile and ethnicity	9%	9%	82%	Selected in round 1			yes
**Indicator #46 (novel)**							
Proportion of women adhering to their treatment	36%	9%	55%	67%	0%	33%	no
**Indicator #46 disaggregated (novel)**							

Building blocks and indicators	ROUND1			ROUND 2			Selected?
	Reject	Indifferent	Select	Reject	Indifferent	Select	
Disaggregated by wealth quintile and ethnicity	36%	9%	55%	75%	0%	25%	no
**Indicator #47 (novel)**							
Proportion of breast cancer patients in high-risk groups who have been offered genetic counselling and testing for germline BRCA1 and BRCA2 mutations	36%	9%	55%	50%	0%	50%	no
**Indicator #47 disaggregated (novel)**							
Disaggregated by wealth quintile and ethnicity	36%	9%	55%	50%	0%	50%	no
**Indicator #48 (novel)**							
Proportion of breast cancer patients who receive *palliative care*	10%	0%	90%	Selected in round 1			yes
**Indicator #48 disaggregated (novel)**							
Disaggregated by wealth quintile and ethnicity	9%	9%	82%	Selected in round 1			yes
**Indicator #49 (novel)**							
Proportion of women terminally ill with breast cancer who receive end-of-life care	9%	0%	91%	Selected in round 1			yes
**Indicator #49 disaggregated (novel)**							
Disaggregated by wealth quintile and ethnicity	9%	9%	82%	Selected in round 1			yes
**Indicator #50 (novel)**							
Proportion of breast cancer survivors who receive survivorship care	45%	0%	55%	50%	13%	38%	no
**Indicator #50 disaggregated (novel)**							
Disaggregated by wealth quintile and ethnicity	45%	9%	45%	50%	13%	38%	no
**Indicator #51 (existing)**							
Self-reported satisfaction with breast surgery: breast conserving surgery and mastectomy	36%	9%	55%	50%	13%	38%	no
**Indicator #51 disaggregated (novel)**							

Building blocks and indicators	ROUND1			ROUND 2			Selected?
	Reject	Indifferent	Select	Reject	Indifferent	Select	
Disaggregated by wealth quintile and ethnicity	36%	18%	45%	50%	13%	38%	no
**Indicator #52 (novel)**							
Proportion of women who were offered breast reconstruction immediately after their mastectomy, excluding inflammatory breast cancer cases	45%	9%	45%	50%	13%	38%	no
**Indicator #52 disaggregated (novel)**							
Disaggregated by wealth quintile and ethnicity	45%	9%	45%	50%	0%	50%	no
**Indicator #53 (novel)**							
Self-reported satisfaction of breast cancer patients with their diagnosis journey: mammogram, ultrasound and breast biopsies	45%	0%	55%	38%	0%	63%	no
**Indicator #53 disaggregated (novel)**							
Disaggregated by wealth quintile and ethnicity	45%	9%	45%	43%	14%	43%	no
**Indicator #54 (novel)**							
Self-reported satisfaction of breast cancer patients with their treatment	55%	9%	36%	78%	11%	11%	no
**Indicator #54 disaggregated (novel)**							
Disaggregated by wealth quintile and ethnicity	45%	9%	45%	75%	0%	25%	no
**Indicator #55 (novel)**							
Self-reported satisfaction of breast cancer patients with the palliative/supportive care received throughout their cancer journey	27%	0%	73%	13%	13%	75%	no
**Indicator #55 disaggregated (novel)**							
Disaggregated by wealth quintile and ethnicity	27%	9%	64%	13%	25%	63%	no
**Indicator #56 (novel)**							
Proportion of breast cancer patients who were followed by one or more of the following during their treatment: a psychologist, a social worker, a dietitian, an exercise physiologist, a genetic counsellor, an occupational therapist, a physiotherapist, and a specialist palliative care.	45%	0%	55%	44%	22%	33%	no
**Indicator #56 disaggregated (novel)**							

Building blocks and indicators	ROUND1			ROUND 2			Selected?
	Reject	Indifferent	Select	Reject	Indifferent	Select	
Disaggregated by wealth quintile and ethnicity	45%	9%	45%	50%	13%	38%	no
**Indicator #57 (novel)**							
Proportion of breast cancer patients who received integrative therapies to help manage stress, anxiety and depression such as music therapy, meditation, stress management, yoga, relaxation, and massage	36%	9%	55%	44%	11%	44%	no
**Indicator #57 disaggregated (novel)**							
Disaggregated by wealth quintile and ethnicity	50%	10%	40%	50%	13%	38%	no
**Indicator #58 (novel)**							
Proportion of breast cancer patients who received integrative therapies to help reduce chemotherapy-induced nausea and vomiting such as acupuncture and acupressure	36%	9%	55%	56%	22%	22%	no
**Indicator #58 disaggregated (novel)**							
Disaggregated by wealth quintile and ethnicity	40%	0%	60%	63%	13%	25%	no
**2. Health financing**							
Structural							
**Indicator #59 (novel)**							
The NCP or NCDP addresses costs of implementation of the breast cancer strategy	25%	0%	75%	10%	10%	80%	yes
**Indicator #60 (existing)**							
Total government health expenditure per capita	42%	8%	50%	60%	10%	30%	no
**Indicator #61 (existing)**							
Share of government spending and out-of-pocket payment out of the total spending on health per capita	25%	0%	75%	10%	0%	90%	yes
**Indicator #62 (novel)**							
Total government spending on health compared with military as a percentage of GDP	25%	8%	67%	50%	20%	30%	no
**Indicator #63 (existing)**							

Building blocks and indicators	ROUND1			ROUND 2			Selected?
	Reject	Indifferent	Select	Reject	Indifferent	Select	
Proportion of total health expenditure dedicated to cancer	33%	8%	58%	30%	30%	40%	no
**Indicator #64 (existing)**							
The state has a *social health insurance system*	8%	8%	83%	Selected in round 1			yes
Process							
**Indicator #65 (novel)**							
The *social health insurance system* covers diagnostic services for breast cancer (i.e., biopsy, mammogram and ultrasound)	18%	9%	73%	20%	0%	80%	yes
**Indicator #66 (novel)**							
The *social health insurance system* covers breast cancer treatment (i.e., hormone therapy and chemotherapy)	10%	10%	80%	Selected in round 1			yes
**Indicator #67 (novel)**							
The *social health insurance system* covers radiotherapy for breast cancer	10%	10%	80%	Selected in round 1			yes
**Indicator #68 (novel)**							
The *social health insurance system* covers palliative care for breast cancer	10%	10%	80%	Selected in round 1			yes
Outcome							
**Indicator #69 (novel)**							
Proportion of breast cancer patients who benefit from the *social health insurance system*	17%	8%	75%	10%	20%	70%	no
**Indicator #69 disaggregated (novel)**							
Disaggregated by wealth quintile, ethnicity *and migration status*	25%	0%	75%	20%	0%	80%	yes
**Indicator #70 (novel)**							
Proportion of costs covered for breast cancer care by the *social health insurance system*	10%	10%	80%	Selected in round 1			yes
**Indicator #71 (novel)**							
Proportion of breast cancer patients with catastrophic health expenditure	17%	17%	67%	20%	20%	60%	no
**Indicator #71 disaggregated (novel)**							

Building blocks and indicators	ROUND1			ROUND 2			Selected?
	Reject	Indifferent	Select	Reject	Indifferent	Select	
Disaggregated by wealth quintile and ethnicity	25%	8%	67%	22%	11%	67%	no
**3. Medicines**							
Structural							
**Indicator #72 (existing)**							
The Constitution, Bill of Rights or other statute recognises the right to access essential medicines	15%	15%	69%	20%	20%	60%	no
**Indicator #73 (existing)**							
There is an official national medicines policy to provide access to essential medicines	15%	0%	85%	Selected in round 1			yes
**Indicator #74 (novel)**							
The NCP or NCDP mentions breast cancer medicines included in the WHO Essential Medicines List	15%	0%	85%	Selected in round 1			yes
Process							
**Indicator #75 (existing)**							
Coverage status of medicines for metastatic breast cancer *that are included in the WHO Essential Medicines List*: not approved, approved but not covered, approved and covered	18%	18%	64%	33%	0%	67%	no
**Indicator #76 (novel)**							
Proportion of breast cancer medicines *included in the WHO Essential Medicines List* that are available in the country and covered by public funding	33%	25%	42%	10%	10%	80%	yes
**Indicator #77 (novel)**							
Proportion of generics in the pharmaceutical market for breast cancer medicines	25%	8%	67%	20%	10%	70%	no
Outcome							
**Indicator #78 (novel)**							
Proportion of hospitals with breast cancer medicines shortage	23%	15%	62%	20%	30%	50%	no
**Indicator #78 disaggregated (novel)**							
Disaggregated by geography	31%	8%	62%	11%	33%	56%	no
**Indicator #79 (novel)**							
Proportion of hospitals with palliative medicines shortage	15%	8%	77%	10%	10%	80%	yes
**Indicator #79 disaggregated (novel)**							

Building blocks and indicators	ROUND1			ROUND 2			Selected?
	Reject	Indifferent	Select	Reject	Indifferent	Select	
Disaggregated by geography	15%	8%	77%	0%	11%	89%	yes
**4. Health workforce**							
Structural							
**Indicator #80 (existing)**							
The state has a national health workforce strategy	23%	0%	77%	11%	0%	89%	yes
**Indicator #81 (novel)**							
The state law includes provision for adequate remuneration of doctors *and* nurses, *including* oncologists	23%	8%	69%	30%	10%	60%	no
**Indicator #82 (novel)**							
The state's workforce policies or programmes provide incentives to promote stationing in rural areas of doctors, nurses and oncologists	23%	0%	77%	20%	10%	70%	no
**Indicator #83 (novel)**							
Human rights are a compulsory part of the national curriculum for the training of doctors, nurses and oncologists	38%	0%	62%	22%	0%	78%	no
Process							
**Indicator #84 (existing)**							
Median time spent for a new consultation, as reported by oncologists	46%	0%	54%	40%	20%	40%	no
**Indicator #85 (existing)**							
Median job satisfaction score of oncologists	58%	17%	25%	80%	20%	0%	no
Outcome							
**Indicator #86 (existing)**							
Prevalence of certified oncologists per 1,000,000 population	15%	0%	85%	Selected in round 1			yes
**Indicator #86 disaggregated (novel)**							

Building blocks and indicators	ROUND1			ROUND 2			Selected?
	Reject	Indifferent	Select	Reject	Indifferent	Select	
Disaggregated by geography	15%	0%	85%	Selected in round 1			yes
**5. Health information**							
Structural							
**Indicator #87 (novel)**							
The state law requires protection of the confidentiality of personal health data	31%	0%	69%	11%	11%	78%	no
**Indicator #88 (novel)**							
The state law allows for *controlled* exceptions to the restrictions of sharing personal health data for epidemiological research *by non-commercial organisations*	31%	8%	62%	30%	10%	60%	no
**Indicator #89 (novel)**							
The state law requires cancer registration	33%	0%	67%	33%	0%	67%	no
**Indicator #90 (existing)**							
The state law requires informed consent to treatment and other health interventions	15%	0%	85%	Selected in round 1			yes
**Indicator #91 (novel)**							
The NCP or NCDP *plans for* cancer research, *independent from pharmaceutical companies*	15%	15%	69%	20%	20%	60%	no
**Indicator #92 (novel)**							
The NCP or NCDP protects the right to seek and receive health information	15%	0%	85%	Selected in round 1			yes
**Indicator #93 (novel)**							

Building blocks and indicators	ROUND1			ROUND 2			Selected?
	Reject	Indifferent	Select	Reject	Indifferent	Select	
The NCP or NCDP addresses communication of information throughout the pathway of care for breast cancer, from screening through to referral, diagnosis, treatment options and palliative care	15%	0%	85%	Selected in round 1			yes
**Indicator #94 (novel)**							
The NCP or NCDP addresses the needs of patients from culturally and linguistically diverse backgrounds	15%	0%	85%	Selected in round 1			yes
**Indicator #95 (novel)**							
The NCP or NCDP addresses participation of patients in decisions that affect them	8%	0%	92%	Selected in round 1			yes
**Indicator #96 (novel)**							
The NCP or NCDP addresses awareness of breast self-examination	31%	8%	62%	22%	11%	67%	no
**Indicator #97 (novel)**							
There is a charter of breast cancer patients' rights freely available and accessible to everyone	38%	8%	54%	11%	22%	67%	no
Process							
**Indicator #98 (novel)**							
There is a national campaign to *raise awareness about* the screening programme	27%	18%	55%	33%	0%	67%	no
**Indicator #99 (novel)**							
The screening programme is implemented through community outreach activities	50%	0%	50%	67%	22%	11%	no
**Indicator #100 (novel)**							
The screening programme includes information on the potential disadvantages and benefits of mammographic screening	25%	8%	67%	13%	13%	75%	no
**Indicator #101 (novel)**							
There is a national strategy to raise awareness on breast self-examination	45%	9%	45%	67%	11%	22%	no

Building blocks and indicators	ROUND1			ROUND 2			Selected?
	Reject	Indifferent	Select	Reject	Indifferent	Select	
**Indicator #102 (novel)**							
There is a national breast cancer patients association *independent from the pharmaceutical industry*	46%	15%	38%	20%	10%	70%	no
**Indicator #103 (novel)**							
The personnel in the breast unit are trained to communicate information on diagnosis and treatment options, including side effects and survival, repeatedly, verbally and in writing, in a comprehensive and easily understandable form to patients	15%	0%	85%	Selected in round 1			yes
**Indicator #104 (novel)**							
The personnel in the breast unit presents all options available to the patient beside a mastectomy, and explains in an easily understandable way psychological distress that may be associated with a mastectomy	15%	0%	85%	Selected in round 1			yes
**Indicator #105 (novel)**							
The personnel in the breast unit discusses breast reconstruction techniques individually taking into account anatomic, treatment- and patient-related factors and preferences	15%	0%	85%	Selected in round 1			yes
**Indicator #106 (novel)**							
The breast unit website or other official source of information includes fertility issues and fertility-preservation techniques	31%	15%	54%	50%	20%	30%	no
**Indicator #107 (novel)**							
The breast cancer unit uses trained interpreters when communicating with patients from culturally and linguistically diverse background	15%	0%	85%	Selected in round 1			yes
**Indicator #108 (novel)**							
The breast unit has a participatory process in place to include patients into decisions that affect them individually	15%	0%	85%	Selected in round 1			yes
**Indicator #109 (novel)**							
The breast unit directs patients to reliable, patient-centred websites	31%	8%	62%	30%	30%	40%	no
**Indicator #110 (novel)**							
There is a state funded cancer registry	8%	25%	67%	0%	22%	78%	no
**Indicator #111 (novel)**							

Building blocks and indicators	ROUND1			ROUND 2			Selected?
	Reject	Indifferent	Select	Reject	Indifferent	Select	
Number of treaty-based grounds of discrimination the cancer registry collects data on: sex; ethnic origin, race, or colour; age; disability; language; religion; national origin; socioeconomic status, social status, social origin, or birth; civil status; political status, or political or other opinion; and property; as well as non treaty-based grounds: people living in rural areas, sexual orientation, gender	18%	18%	64%	40%	20%	40%	no
Outcome							
**Indicator #112 (novel)**							
Proportion of women aged 50-69 who are aware of the importance of breast cancer screening	50%	0%	50%	38%	38%	25%	no
**Indicator #112 disaggregated (novel)**							
Disaggregated by wealth quintile and ethnicity	50%	8%	42%	43%	29%	29%	no
**Indicator #113 (novel)**							
Proportion of women who had their mammography screening in the last *24 months* and were informed of the potential disadvantages and benefits of mammographic screening	42%	8%	50%	33%	0%	67%	no
**Indicator #113 disaggregated (novel)**							
Disaggregated by wealth quintile and ethnicity	42%	8%	50%	25%	0%	75%	no
**Indicator #114 (novel)**							
Proportion of breast cancer patients who feel they have received sufficient, comprehensive and easily understandable information, including on treatment side effects and survival, to be involved in decisions about their care	15%	8%	77%	10%	0%	90%	yes
**Indicator #114 disaggregated (novel)**							
Disaggregated by wealth quintile, ethnicity, *and language*	15%	8%	77%	11%	0%	89%	yes
**Indicator #115 (novel)**							
Proportion of women from culturally or linguistically diverse backgrounds who were offered an interpreter to communicate during their breast cancer journey	23%	8%	69%	22%	0%	78%	no
**Indicator #115 disaggregated (novel)**							
Disaggregated by wealth quintile and ethnicity	25%	0%	75%	25%	0%	75%	no
**Indicator #116 (novel)**							
Proportion of breast cancer patients who feel they have been involved in decisions about their care	15%	0%	85%	Selected in round 1			yes
**Indicator #116 disaggregated (novel)**							

Building blocks and indicators	ROUND1			ROUND 2			Selected?
	Reject	Indifferent	Select	Reject	Indifferent	Select	
Disaggregated by wealth quintile and ethnicity	15%	8%	77%	22%	11%	67%	no
**Indicator #117 (novel)**							
Proportion of breast cancer patients who feel they have received sufficient, comprehensive and easily understandable information about their mastectomy	15%	8%	77%	22%	0%	78%	no
**Indicator #117 disaggregated (novel)**							
Disaggregated by wealth quintile and ethnicity	15%	8%	77%	22%	0%	78%	no
**Indicator #118 (novel)**							
Proportion of breast cancer patients who feel they have been involved in making the decision to undergo a mastectomy	23%	0%	77%	22%	0%	78%	no
**Indicator #118 disaggregated (novel)**							
Disaggregated by wealth quintile and ethnicity	23%	8%	69%	22%	0%	78%	no
**Indicator #119 (novel)**							
Proportion of premenopausal patients who received sufficient, comprehensive and easily understandable information about fertility issues and fertility-preservation techniques before receiving their treatment	23%	8%	69%	0%	33%	67%	no
**Indicator #119 disaggregated (novel)**							
Disaggregated by wealth quintile and ethnicity	23%	8%	69%	11%	22%	67%	no
**Indicator #120 (novel)**							
Proportion of women with breast cancer who felt that they received sufficient information at all stages of their cancer journey, from screening through to referral, diagnosis, treatment options and palliative care	23%	8%	69%	30%	10%	60%	no
**Indicator #120 disaggregated (novel)**							
Disaggregated by wealth quintile and ethnicity	23%	8%	69%	33%	11%	56%	no
**Indicator #121 (novel)**							

Building blocks and indicators	ROUND1			ROUND 2			Selected?
	Reject	Indifferent	Select	Reject	Indifferent	Select	
Proportion of women who are aware of the importance of breast self-examination	42%	0%	58%	38%	13%	50%	no
**Indicator #121 disaggregated (novel)**							
Disaggregated by wealth quintile and ethnicity	42%	8%	50%	38%	0%	63%	no
**Indicator #122 (novel)**							
Proportion of women regularly undertaking breast self-examination	42%	8%	50%	38%	25%	38%	no
**Indicator #122 disaggregated (novel)**							
Disaggregated by wealth quintile and ethnicity	42%	8%	50%	38%	13%	50%	no
**Indicator #123 (novel)**							
Self-reported reason for women diagnosed with locally advanced or advanced breast cancer for not seeking care at an earlier stage of the disease	42%	8%	50%	13%	38%	50%	no
**Indicator #123 disaggregated (novel)**							
Disaggregated by wealth quintile and ethnicity	42%	8%	50%	25%	25%	50%	no
**6. Governance / Leadership**							
Structural							
**Indicator #124 (existing)**							
The Constitution, Bill of Rights, or other statute recognises the right to health	17%	8%	75%	20%	0%	80%	yes
**Indicator #125 (novel)**							
The state has transparent rules on lobbying, including a public lobbying register	31%	15%	54%	11%	33%	56%	no
**Indicator #126 (novel)**							

Building blocks and indicators	ROUND1			ROUND 2			Selected?
	Reject	Indifferent	Select	Reject	Indifferent	Select	
The state has an NCP or NCDP addressing breast cancer specifically	31%	15%	54%	30%	20%	50%	no
**Indicator #127 (novel)**							
The NCP or NCDP addresses accountability of the state and health institutions	8%	15%	77%	0%	20%	80%	yes
**Indicator #128 (novel)**							
The NCP or NCDP addresses vulnerable populations such as ethnic minorities, refugees, homosexuals, and LBGQ+	17%	8%	75%	22%	11%	67%	no
**Indicator #129 (novel)**							
The NCP or NCDP explicitly recognises the right to health	33%	8%	58%	40%	10%	50%	no
**Indicator #130 (novel)**							
The NCP or NCDP includes a protection against discrimination	23%	0%	77%	11%	0%	89%	yes
**Indicator #131 (novel)**							
The NCP or NCDP includes an explicit commitment to universal access to cancer services and treatment	15%	0%	85%	Selected in round 1			yes
**Indicator #132 (novel)**							
The NCP or NCDP includes a set of targets and progress indicators *specific to breast cancer*	23%	0%	77%	10%	0%	90%	yes
**Indicator #133 (novel)**							

Building blocks and indicators	ROUND1			ROUND 2			Selected?
	Reject	Indifferent	Select	Reject	Indifferent	Select	
The state undertook a health impact assessment before adopting the national cancer plan	23%	0%	77%	20%	10%	70%	no
**Indicator #134 (novel)**							
The health impact assessment explicitly included the right to health	25%	8%	67%	30%	0%	70%	no
**Indicator #135 (novel)**							
The NCP or NCDP includes a requirement for the participation of marginalised groups	23%	0%	77%	22%	0%	78%	no
**Indicator #136 (novel)**							
The Ministry of Health collaborates with the Ministries of Employment, Education, or Finance on cancer policy	25%	8%	67%	30%	30%	40%	no
Process							
**No indicator**							
Outcome							
**Indicator #137 (existing)**							
State's corruption index	50%	0%	50%	40%	10%	50%	no
**Indicator #138 (existing)**							
State's rule of law index	64%	0%	36%	50%	20%	30%	no
**7. Underlying determinants of breast cancer**							
Structural							
**Indicator #139 (novel)**							

Building blocks and indicators	ROUND1			ROUND 2			Selected?
	Reject	Indifferent	Select	Reject	Indifferent	Select	
The state is a party to the Framework Convention on Tobacco Control	42%	0%	58%	25%	13%	63%	no
**Indicator #140 (existing)**							
The state has an operational policy, strategy or action plan to reduce the burden of tobacco use	33%	0%	67%	13%	13%	75%	no
**Indicator #141 (existing)**							
The state has an operational policy, strategy or action plan to reduce overweight and obesity	33%	0%	67%	25%	13%	63%	no
**Indicator #142 (existing)**							
The state has an operational policy, strategy or action plan to reduce physical inactivity and/or promote physical activity	33%	0%	67%	25%	13%	63%	no
**Indicator #143 (existing)**							
The state has an operational policy, strategy or action plan to reduce the harmful use of alcohol	33%	0%	67%	29%	14%	57%	no
Process							
**No indicator**							
Outcome							
**Indicator #144 (novel)**							
The country air pollution is below the WHO recommended annual mean (10 µg/m3 for fine particulate matter PM2.5 and 20 µg/m3 for coarse particulate matter PM10)	33%	8%	58%	25%	25%	50%	no
**Indicator #145 (existing)**							
Proportion of adult women who are smokers	33%	8%	58%	33%	22%	44%	no
**Indicator #146 (existing)**							
Proportion of adult women who are overweight and obese	36%	9%	55%	33%	22%	44%	no
**Indicator #147 (existing)**							

Building blocks and indicators	ROUND1			ROUND 2			Selected?
	Reject	Indifferent	Select	Reject	Indifferent	Select	
Prevalence of hazardous drinking among women	33%	0%	67%	33%	22%	44%	no
**8. Redress**							
Structural							
**Indicator #148 (existing)**							
There is an accessible pre-judicial mechanism to lodge complaints alleging breach of obligations connected to the right to health	17%	0%	83%	Selected in round 1			yes
Process							
**Indicator #149 (novel)**							
Number of court cases litigated on cancer in a given year	67%	17%	17%	90%	0%	10%	no
**Indicator #150 (novel)**							
Proportion of class actions among the cases litigated on cancer in a given year	46%	15%	38%	80%	10%	10%	no
**Indicator #151 (novel)**							
The breast unit has a formal complaints mechanism for patients	8%	8%	85%	Selected in round 1			yes
**Indicator #151 disaggregated (novel)**							
Disaggregated by geography	15%	8%	77%	50%	0%	50%	no
Outcome							
**No indicator**							
**ADDITIONAL INDICATORS SUGGESTED IN ROUND 1, FOR SELECTION IN ROUND 2**							
**Indicator #152 (novel) - to replace indicator #13**							

Building blocks and indicators	ROUND1			ROUND 2			Selected?
	Reject	Indifferent	Select	Reject	Indifferent	Select	
Proportion of the population at risk participating in the screening programme	NA			14%	0%	86%	yes
**Indicator #153 (novel)**							
Prevalence of certified nurses per 1,000,000 population	NA			13%	0%	88%	yes
**Indicator #154 (existing)**							
The State has ratified key human rights treaties recognising the right to health	NA			13%	0%	88%	yes

End of document

Words in italic and underlined show reformulation as per the panel's suggestions.
